# Novel roles of RTN4 and CLIMP-63 in regulating mitochondrial structure, bioenergetics and apoptosis

**DOI:** 10.1038/s41419-022-04869-8

**Published:** 2022-05-04

**Authors:** Rachel J. Carter, Mateus Milani, Alison J. Beckett, Shiyu Liu, Ian A. Prior, Gerald M. Cohen, Shankar Varadarajan

**Affiliations:** 1 Departments of Molecular and Clinical Cancer Medicine, Institute of Systems, Molecular and Integrative Biology, University of Liverpool, Liverpool, L69 3GE UK; 2 Molecular Physiology and Cell Signaling, Institute of Systems, Molecular and Integrative Biology, University of Liverpool, Liverpool, L69 3GE UK

**Keywords:** Cell biology, Cancer

## Abstract

The recruitment of DRP1 to mitochondrial membranes prior to fission is facilitated by the wrapping of endoplasmic reticulum (ER) membranes around the mitochondria. To investigate the complex interplay between the ER membranes and DRP1 in the context of mitochondrial structure and function, we downregulate two key ER shaping proteins, RTN4 and CLIMP-63, and demonstrate pronounced mitochondrial hyperfusion and reduced ER-mitochondria contacts, despite their differential regulation of ER architecture. Although mitochondrial recruitment of DRP1 is unaltered in cells lacking RTN4 or CLIMP-63, several aspects of mitochondrial function, such as mtDNA-encoded translation, respiratory capacity and apoptosis are significantly hampered. Further mechanistic studies reveal that CLIMP-63 is required for cristae remodeling (OPA1 proteolysis) and DRP1-mediated mitochondrial fission, whereas both RTN4 and CLIMP-63 regulate the recruitment of BAX to ER and mitochondrial membranes to enable cytochrome *c* release and apoptosis, thereby performing novel and distinct roles in the regulation of mitochondrial structure and function.

## Introduction

The dynamic nature of mitochondria is facilitated by several fusion and fission GTPases, all of which are integral for the maintenance of mitochondrial structure and function. Defects in mitochondrial membrane dynamics have been implicated in pathophysiological conditions, including poor brain development, optic atrophy, cardiomyopathy and neurodegenerative diseases [1–3], thus emphasising the need to understand how mitochondrial fission and fusion events are regulated. The fusion GTPases, mitofusins (MFN1/2) and OPA1 reside on mitochondrial membranes, whereas the fission GTPase, DRP1 is translocated from the cytosol to the outer mitochondrial membrane (OMM) to induce mitochondrial fission [4]. The recruitment of DRP1 to mitochondria occurs following the marking (wrapping) of mitochondrial constriction sites by membranes of the endoplasmic reticulum (ER) [5]. ER architecture comprises of two domains: a reticular network of tubules and flattened sheets, which are maintained by proteins of the reticulon (RTN) family and CLIMP-63, respectively [6, 7]. A shift in the balance between ER tubules and sheets could potentially alter how the ER membranes communicate with mitochondria, *via* ER-mitochondria contact sites (ERMCS), thus affecting mitochondrial structure as well as function.

Maintenance of mitochondrial structure is critical for diverse cellular processes, ranging from bioenergetics and metabolism to intracellular signalling and apoptosis. Several mitochondrial-resident proteins, encoded either by nuclear or mitochondrial DNA (mtDNA), are key components of the electron transport chain (ETC) and oxidative phosphorylation, which maintain mitochondrial membrane potential (*via* H^+^ gradient) and ATP production. The expression levels of mtDNA-encoded proteins, and consequently their associated functions could be severely disrupted by ineffective maintenance of mitochondrial and/or ERMCS structure [8, 9]. Moreover, the mitochondrial fusion GTPase, MFN2 tethers ER and mitochondrial membranes to form ERMCS [10], which in turn marks mitochondrial constriction sites for the recruitment of DRP1. In addition to the induction of mitochondrial fission, membrane recruitment of DRP1 also facilitates the release of cytochrome *c* from the mitochondria to the cytosol at the onset of apoptosis [11, 12]. Cytochrome *c*, generally restricted to the mitochondrial cristae, is redistributed to the intermembrane space following the proteolytic processing of OPA1 [13] and released from the mitochondria by a process called mitochondrial outer membrane permeabilisation (MOMP). MOMP is accomplished by the mitochondrial translocation of BAX and its ensuing oligomerisation (pore formation) with active BAK [14, 15], subsequently activating the initiator and effector caspases, thus culminating in the final stages of the intrinsic apoptotic pathway.

In this report, we identify and characterise distinct roles for two major ER shaping proteins, RTN4 and CLIMP-63, in the execution of mitochondrial fragmentation, cristae remodelling (OPA1 proteolysis), BAX translocation, MOMP, and apoptosis. Furthermore, we show that the depletion of RTN4 and CLIMP-63 compromises mitochondrial respiratory capabilities, revealing new molecular insights into the mechanisms underlying ER membrane dynamics and cancer cell death.

## Results

### DRP1 differentially regulates mitochondrial morphology in several cell lines

**Fig. 1:**
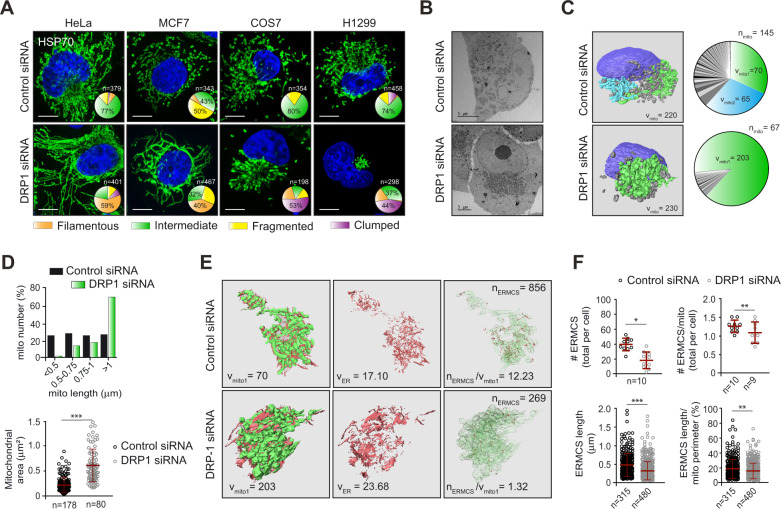
Depletion of DRP1 alters mitochondrial morphology that accompanies a reduction in ER-mitochondria contacts. **A** The indicated cell lines were transfected with a non-targeting control or DRP1 siRNA for 72 h, immunostained for HSP70 (green) and mitochondrial morphologies quantified as filamentous, intermediate, fragmented or clumped in the indicated number of cells (*n*), and plotted in pie charts here, and as bar graphs in Supplementary Fig. 13. Scale bars are 10 μm. **B** TEM images of H1299 cells transfected with control or DRP1 siRNA for 72 h. Scale bars are 5 μm. **C** Rendered 3View images of the nuclei (purple), and mitochondria (green, light blue or different shades of grey) in H1299 cells transfected as in (**B)**. Pie charts to the right depict the total number of mitochondria (*n*_mito_) in each rendered cell. V_mito_, V_mito1_ and V_mito 2_ denote the volumes (μm^3^) of specific mitochondria, colour-coded according to the adjacent images. **D** Mitochondrial length (μm), number (%) and area (μm^3^) were assessed from TEM images of at least 10 cells per siRNA. The number of mitochondria counted (*n*) are specified below the *x*-axis. **E** The endoplasmic reticulum (red) wrapping around the chosen mitochondrion (in green; from **C**) was rendered to compare volumes (μm^3^) of the mitochondrion (V_mito1_) and associated ER membranes (V_ER_). The far-right panel shows ER-mitochondria contact sites (ERMCS; red dots) juxtaposed on the mitochondrial skeleton (translucent green), along with the number of ERMCS, both absolute (n_ERMCS_) and normalised (*n*_ERMCS_/V_mito1_). **F** ERMCS were quantified from the same TEM images as in (**D)** and values plotted to show the total number per cell (#ERMCS/cell), the average number per mitochondrion (#ERMCS/mito/cell) and the length (μm), both per cell and relative to mitochondrial perimeter (%). For the top two graphs, each dot represents the total ERMCS per cell in the indicated number of cells (*n*); for the bottom two graphs, each dot represents one ERMCS with *n* values detailed below the *x* axes. All were quantified from 10 cells per siRNA, from 3 independent experiments. Data are presented as mean ± SD; *p ⩽ 0.05, **p ⩽ 0.005, ***p ⩽ 0.001 (Mann-Whitney *U* test).

It has been well-documented that mitochondria in different cell lines exhibit variations with respect to their morphology, ranging from being highly filamentous to extensively fragmented. Silencing the expression of DRP1, a critical fission GTPase, resulted in mitochondrial hyperfusion, broadly characterised by a reduction in the percentage of cells with mitochondria that exhibited an intermediate morphology, i.e. neither completely filamentous nor fragmented (Fig. 1A). More specifically, mitochondria in HeLa and MCF7 cells assumed a filamentous morphology following DRP1 downregulation, in stark contrast to the juxtanuclear clustering/clumping of mitochondria evident in ~50 % of the DRP1-depleted COS7 and H1299 cells, despite a consistent increase in mitochondrial length and reduced mitochondrial number in all cell lines (Fig. 1A, B and Supplementary Figs. 1A, B and 13). To understand whether such clumped mitochondria were in fact interconnected, we employed serial block-face scanning electron microscopy (3View microscopy) on sequential sections covering the entire volume of the chosen cells. The acquired images were used to render organelles of interest (nucleus in purple and mitochondria in blue, green or different shades of grey) and modelled as 3D configurations (Fig. 1C). Although the combined volume of all mitochondria (V_mito_) in cells transfected with control siRNA and DRP1 siRNA were roughly similar, at 220 and 230 μm^3^, respectively (Fig. 1C), the number of individual mitochondria (n_mito_) was considerably reduced following DRP1 depletion: 67, compared to 145 in the control cell (Fig. 1C), supporting a loss of mitochondrial fission in the absence of DRP1. Furthermore, the two fused mitochondria in the control cell (green and blue), each representing ~30% of the total mitochondrial volume (with the remaining 40% occupied by 143 smaller/fragmented mitochondria, in grey) were strikingly different from the single hyperfused mitochondrion (green) that occupied an astonishing 88% of the total mitochondrial volume in the DRP1-depleted cell (Fig. 1C). In addition to the increased mitochondrial length and reduced mitochondrial number (Fig. 1D and Supplementary Fig. 1A), DRP1 downregulation also increased mitochondrial area (Fig. 1D), further confirming that the loss of DRP1 resulted in a marked reduction in mitochondrial fission, which manifested in cells as mitochondrial hyperfusion.

### DRP1 is critical for the maintenance of ER-mitochondria contacts

Since ER tubules wrap around mitochondria to mark constriction sites and facilitate DRP1-mediated fission [5], we wished to test whether a loss of DRP1 would impact the ability of the ER (rendered in red) to establish contacts with a mitochondrion (rendered in green; from Fig. 1C) (Fig. 1E). In the cell lacking DRP1, the tubular architecture of the ER membranes acquired a sheet-like morphology, with increased ER volume (V_ER_) (23.7 *versus* 17.1 μm^3^ in the control cell) and exhibited a marked reduction in the extent of wrapping around the mitochondrion (Fig. 1E). This was further confirmed by reduced numbers (~9-fold) of ERMCS (n_ERMCS_; red dots superimposed on a green or blue translucent mitochondrial skeleton), following DRP1 downregulation (Fig. 1E and Supplementary Fig. 2). To avoid drawing conclusions based on calculations from a single cell, we measured the number and length of ERMCS on at least 3 micrographs acquired per cell, from 10 different cells in control siRNA- and DRP1 siRNA-transfected cells, and report significant decreases in the number and length of ERMCS following DRP1 downregulation (Fig. 1F), thereby confirming the requirement of DRP1 in maintaining ERMCS.

### ER shaping proteins, RTN4 and CLIMP-63 also regulate mitochondrial hyperfusion and ERMCS

**Fig. 2:**
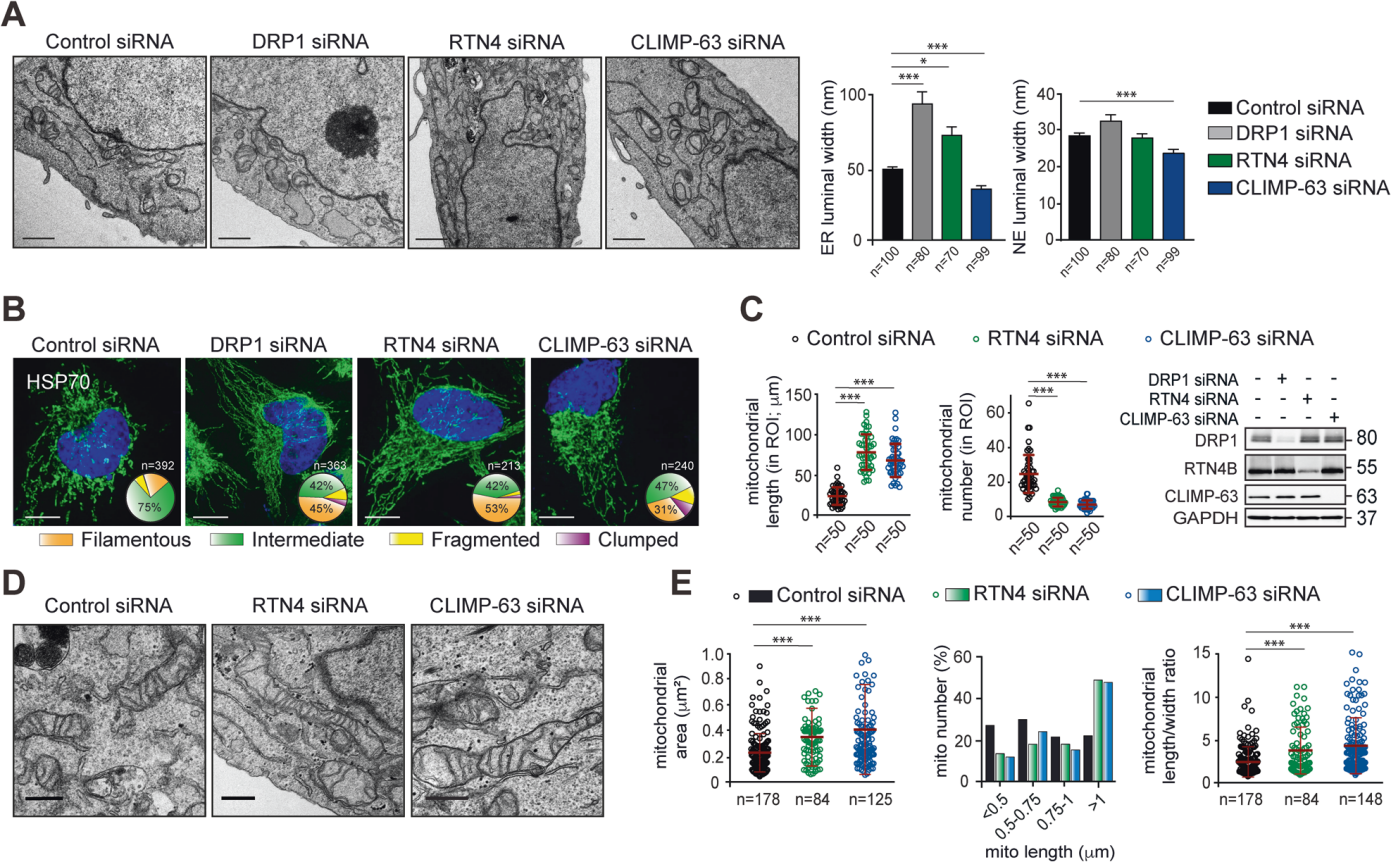
ER shaping proteins, RTN4 and CLIMP-63, regulate ER and mitochondrial morphology. **A** Representative TEM images and graphs, showing ER and nuclear envelope (NE) width in HeLa cells transfected with a non-targeting control, DRP1, RTN4 or CLIMP-63 siRNA for 72 h. The number of ER or NE lumen used for width calculations are denoted as *n* numbers below the *x* axes. **B** HeLa cells were transfected with the indicated siRNAs for 72 h and immunostained for HSP70 (green) to visualise mitochondrial structure. Mitochondrial morphologies were quantified in the indicated numbers of cells per condition, across a minimum of 3 independent experiments, and the data plotted in pie charts here, and as bar graphs in Supplementary Fig. 13. Scale bars are 10 μm. **C** Mitochondrial length (μm) and number were quantified in a defined region of interest (ROI) from images of 50 cells (*n* = 50) acquired as in **B**. Western blots show DRP1, RTN4 and CLIMP-63 knockdown efficiencies for the indicated siRNAs. **D** TEM images of mitochondria in HeLa cells, transfected with the indicated siRNAs for 72 h. Scale bars are 500 nm. **E** Mitochondrial area (μm^3^), length (μm), length/width ratio and number (%) were assessed by calculating these parameters from all mitochondria identified in the TEM images of at least 10 HeLa cells per siRNA. The number of mitochondria counted in each of these conditions are presented as n numbers below the *x* axes. Data are presented as **A**, mean ± SEM, or **C** and **E**, mean ± SD; *p ⩽ 0.05, ***p ⩽ 0.001 (Kruskal-Wallis tests, with Dunn’s multiple comparisons tests).

Since ER membranes play an integral role in mitochondrial constriction and DRP1-mediated fission, we sought to ascertain whether changes in ER membrane morphology could alter mitochondrial structure or function. Therefore, we silenced two ER shaping proteins, RTN4 and CLIMP-63, which are responsible for maintaining the architecture of ER tubules and sheets, respectively [6, 7]. Consistent with their purported functions, silencing of RTN4 and CLIMP-63 respectively enhanced and decreased the luminal width of the ER membranes (Fig. 2A). More strikingly, downregulation of DRP1 significantly increased ER luminal width, to a greater extent than that of RTN4 siRNA, further confirming our earlier findings of enhanced sheet-like ER morphology in cells lacking DRP1 (Fig. 1E). In contrast, the luminal width of the nuclear envelope (NE) remained largely unaltered following downregulation of these proteins, in agreement with previous findings [7], although there was a small yet statistically significant reduction of NE luminal width in cells lacking CLIMP-63 (Fig. 2A). Moreover, changes in ER luminal width did not accompany ER stress and the unfolded protein response (UPR) (Supplementary Fig. 3A), thus confirming that changes in mitochondrial morphology in cells lacking DRP1 or the ER shaping proteins occurred in an UPR-independent manner (Supplementary Figs. 3B and 13).

Next, we assessed whether such changes in ER tubule-sheet dynamics could affect mitochondrial morphology. Surprisingly, downregulation of both RTN4 and CLIMP-63 resulted in the appearance of filamentous mitochondria in HeLa cells, to an extent similar to that of DRP1 siRNA (Fig. 2B and Supplementary Fig. 13). Moreover, silencing of both RTN4 and CLIMP-63 resulted in a drastic reduction in mitochondrial fragmentation in MCF7 and increased mitochondrial clumping in COS7 and H1299 cells (Supplementary Figs. 4A and 13), as previously observed with DRP1 downregulation (Fig. 1A). Such mitochondrial morphological changes accompanied a uniform increase in mitochondrial length and decreased mitochondrial number in all four cell types (Fig. 2C and Supplementary Fig. 4B). Furthermore, TEM images of cells lacking RTN4 and CLIMP-63 revealed hyperfused mitochondria in both HeLa and H1299 cells (Fig. 2D and Supplementary Fig. 4D), which accompanied an increase in mitochondrial area, length and length/width ratio (Fig. 2E), as well as a modest decrease in the number and length of ERMCS (Supplementary Fig. 4E). How ER shaping proteins regulate such striking structural changes in the ER and mitochondria remains unclear, as these effects could not be attributed to changes in ER tubule-sheet dynamics (Fig. 2A) or altered expression levels of ER sheet proteins (KTN-1, TRAP-α) or mitochondrial proteins (HSP70, TOMM20) (Supplementary Fig. 5). Furthermore, no significant changes could be observed in relation to expression levels of proteins involved in the mitochondrial fusion (MFN1, MFN2, OPA1) or fission (MFF, MiD49, MiD51, phosphorylation status of DRP1) machinery (Supplementary Fig. 5).

### Mitochondrial hyperfusion mediated by RTN4 and CLIMP-63 downregulation does not always accompany morphological changes to endosomes or peroxisomes

In addition to its well-characterised role in mitochondrial fission, DRP1 has also been reported to regulate membrane dynamics of other organelles, particularly peroxisomes [16]. In agreement, silencing of DRP1 caused a significant increase in the length of PEX-14-positive peroxisomes, which accompanied a striking reduction in peroxisomal number (Supplementary Figs. 6 and 13). Silencing of RTN4, but not CLIMP-63 resulted in a modest increase in peroxisomal length and decreased peroxisomal number, despite both siRNAs inducing similar levels of mitochondrial hyperfusion (Supplementary Figs. 6 and 13). It is unclear whether such increase in peroxisomal length in cells lacking DRP1 and RTN4 could be attributed to enhanced ER luminal width, which could suggest distinct mechanisms employed by the ER membranes to communicate with different subcellular organelles. Nonetheless, neither DRP1, RTN4 nor CLIMP-63 siRNA resulted in marked changes to EEA1-positive early endosomes (Supplementary Fig. 6).

### Downregulation of RTN4 and CLIMP-63 antagonises several inducers of mitochondrial fragmentation

**Fig. 3:**
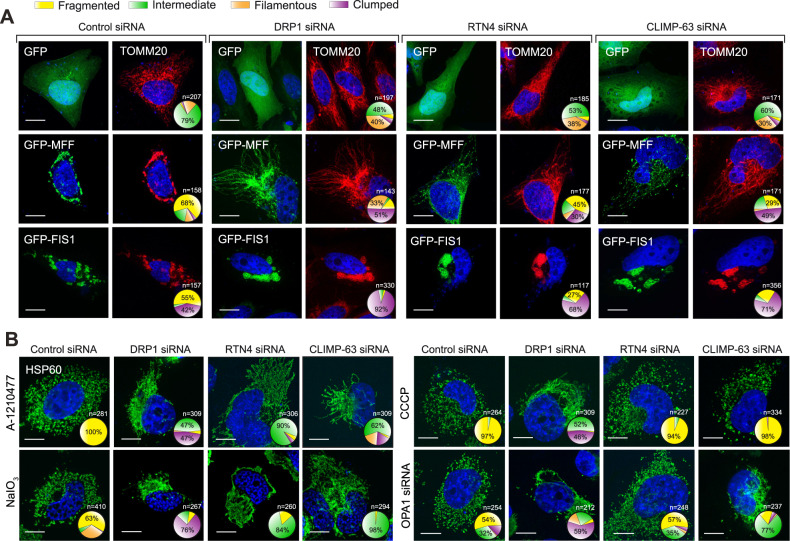
Downregulation of the ER shaping proteins, RTN4 and CLIMP-63 antagonises the effects of several mitochondrial fragmentation inducers. **A** HeLa cells, transfected with a non-targeting control, DRP1, RTN4 or CLIMP-63 siRNA for 72 h, were also transfected with either a GFP, GFP-MFF or GFP-Fis1 plasmid for the final 24 h, then immunostained using an antibody against TOMM20 (red). Mitochondrial morphologies were quantified in the indicated numbers of cells (*n*) per condition, across a minimum of 3 independent experiments, and the data plotted in pie charts here, and as bar graphs in Supplementary Fig. 13. **B** H1299 cells, transfected with a non-targeting control, DRP1, RTN4 or CLIMP-63 siRNA for 72 h, were exposed for 4 h to A-1210477 (10 μM), NaIO_3_ (100 μM), CCCP (20 μM) or co-transfected with OPA1 siRNA, immunostained using an antibody against HSP60 (green) and quantified as in **A**. Scale bars are 10 μm.

To further interrogate the requirement of RTN4 and CLIMP-63 for efficient mitochondrial fragmentation, we sought to test whether the depletion of either of these proteins was able to rescue the effects of mitochondrial fragmentation inducers. The pronounced mitochondrial fragmentation induced by the overexpression of MFF (recruits DRP1 to the mitochondrial fission site) [17] or Fis1 (also involved in the mitochondrial recruitment of DRP1, whilst additionally inhibiting mitochondrial fusion) [18, 19] was diminished upon downregulation of RTN4 or CLIMP-63, to a similar extent as in a DRP-1 deficient background, in both HeLa and H1299 cells (Fig. 3A and Supplementary Figs. 7 and 13). Furthermore, Fis1-mediated mitochondrial fragmentation could be efficiently restored in RTN4- and CLIMP-63-silenced cells by concomitant overexpression of RTN4B and CLIMP-63, respectively (Supplementary Figs. 8 and 13). The regulatory roles of RTN4 and CLIMP-63 were not just restricted to MFF- or Fis1-mediated mitochondrial fragmentation, as cells lacking DRP1, RTN4 or CLIMP-63 antagonised mitochondrial fragmentation mediated by other agents, such as A-1210477 [12] and NaIO_3_ [20] (Fig. 3B and Supplementary Fig. 13). However, these findings could not be replicated with other fragmentation inducers, such as CCCP (in which mitochondrial fragmentation was rescued by the depletion of DRP1 but not RTN4 or CLIMP-63) and OPA1 siRNA (in which mitochondrial fragmentation was rescued by the depletion of DRP1 and CLIMP-63, but not RTN4) (Fig. 3B and Supplementary Fig. 13). Thus, the differential dependence on DRP1, RTN4 and CLIMP-63 of disparate mitochondrial fragmentation inducers implied that these proteins each have differing mechanisms of action in the regulation of mitochondrial membrane dynamics.

### Mitochondrial translocation of DRP1 is largely retained in the absence of RTN4 or CLIMP-63

**Fig. 4:**
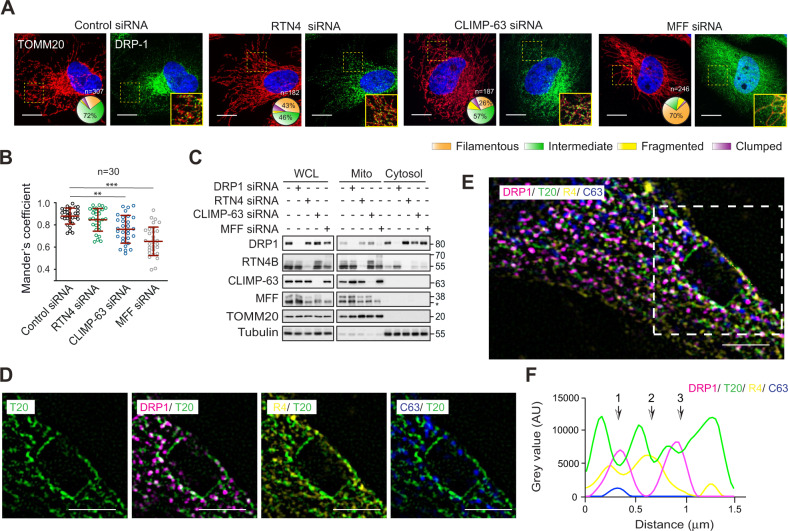
Silencing of ER shaping proteins does not alter mitochondrial recruitment of DRP1. **A** HeLa cells were transfected with either a control, RTN4, CLIMP-63 or MFF siRNA (as a positive control) for 72 h, then immunostained using antibodies against DRP1 (green) and TOMM20 (red). Mitochondrial morphologies were quantified in the indicated numbers of cells (*n*) per condition, across a minimum of 3 independent experiments, and the data plotted in pie charts here, and as bar graphs in Supplementary Fig. 13. Areas demarcated by the dashed yellow boxes are merged and magnified in the rightmost panel of each condition. Scale bars are 10 μm. **B** The extent of colocalisation of DRP1 foci with mitochondria (TOMM20), represented by Mander’s coefficient, was calculated from a defined ROI per cell in 30 different cells per condition. In the graph, each dot represents one ROI. Data are displayed as mean ± SD, **p ⩽ 0.005, ***p ⩽ 0.001 (Kruskal-Wallis test, with Dunn’s multiple comparisons test). **C** Western blots of the indicated proteins in whole cell lysates (WCL), as well as fractions corresponding to mitochondria (Mito) and cytosol, isolated from cells transfected as detailed in **A**. A non-specific band in the MFF blot is denoted with an asterisk (*). **D** HeLa cells were immunostained using antibodies against DRP1 (magenta), RTN4 (R4; yellow), CLIMP-63 (C63; blue) and TOMM20 (T20; green) and subjected to superresolution microscopy. **E** Superresolution image of the cell immunostained in **D**, in which the white dashed square corresponds to the ROI shown in **D**. Scale bars are 2 μm. **F** Relative intensity plots, depicted as Grey value in absorbance units (AU) of the indicated markers, for the dashed line (in orange) drawn within the ROI in **E**. The arrows labelled 1–3 correspond to potential mitochondrial constriction/fission sites.

The ability of DRP1 to induce mitochondrial fission is dependent upon its translocation from the cytosol to its receptors, such as MFF on mitochondrial membranes [17]. Since our results revealed that the ER shaping proteins were as effective as DRP1 in the regulation of mitochondrial fragmentation, we hypothesised that the ER shaping proteins could facilitate the mitochondrial translocation of DRP1, and if so, DRP1 would be retained in the cytosol of cells lacking RTN4 and CLIMP-63. While downregulation of MFF (used as a positive control to diminish DRP1 translocation) drastically reduced the colocalisation of DRP1 with a mitochondrial membrane protein, TOMM20 (assessed by Mander’s coefficient), depletion of RTN4 caused only a modest reduction in the recruitment of DRP1 to mitochondrial membranes (Fig. 4A, B). In contrast, downregulation of CLIMP-63 resulted in a significant reduction in the colocalisation of DRP1 and TOMM20 (Fig. 4A, B), suggesting a potential role for CLIMP-63 in the mitochondrial recruitment of DRP1. To confirm these observations using an independent strategy, we performed subcellular fractionation, which demonstrated a marked reduction of DRP1 in the mitochondrial fraction following MFF depletion, but not following RTN4 or CLIMP-63 downregulation (Fig. 4C). Taken together, our results suggested that mitochondrial translocation of DRP1 may not require RTN4 or CLIMP-63, even though efficient mitochondrial fragmentation could be achieved by DRP1 only in the presence of RTN4 or CLIMP-63.

### RTN4 and CLIMP-63 localise to ERMCS to facilitate mitochondrial fragmentation

Although DRP1 translocation to the mitochondria is not dependent on either RTN4 or CLIMP-63, both RTN4 and CLIMP-63 preferentially localised to the mitochondrial fraction (Fig. 4C) - similar to the mitochondria-resident MFF and unlike cytosolic tubulin - possibly enabling ERMCS formation and mitochondrial fission. The presence of RTN4 and CLIMP-63 at ERMCS was further confirmed both by further fractionation, including isolation of mitochondria-associated membranes (MAM) (Supplementary Fig. 9), and by superresolution lattice structured illumination microscopy (SIM) (Fig. 4D, E). While DRP1 was largely restricted to the cytosol (and the nuclear fraction), with traces found in the mitochondrial fraction, both RTN4 and CLIMP-63 almost exclusively localised to the MAM-enriched fraction (Supplementary Fig. 9). Furthermore, DRP1-positive punctae clearly localised to the ends and/or constriction sites of most mitochondrial filaments (Fig. 4D, E). A dashed line (length 1.5 μm, in orange; Fig. 4E), drawn to assess the intensity profiles of the four markers of interest (DRP1/RTN4/CLIMP-63/TOMM20) along its length, revealed dips in the fluorescence intensity of mitochondria (TOMM20; green) at intervals, potentially indicating three different constriction/fission sites (depicted by numbered arrows in Fig. 4E). Interestingly, the fluorescence intensity of DRP1 (magenta) peaked at sites 1 and 3, and this overlapped with both RTN4 (yellow) and CLIMP-63 (blue) at site 1 and just RTN4 at site 3 (Fig. 4F). The fluorescence intensity of RTN4 additionally peaked at potential constriction site 2 (Fig. 4F). Taken together, these results implied that both RTN4 and CLIMP-63 are physically involved in the execution of mitochondrial constriction and/or fission, independent of DRP1 translocation/mitochondrial recruitment.

### Depletion of ER shaping proteins compromises mitochondrial respiration

**Fig. 5:**
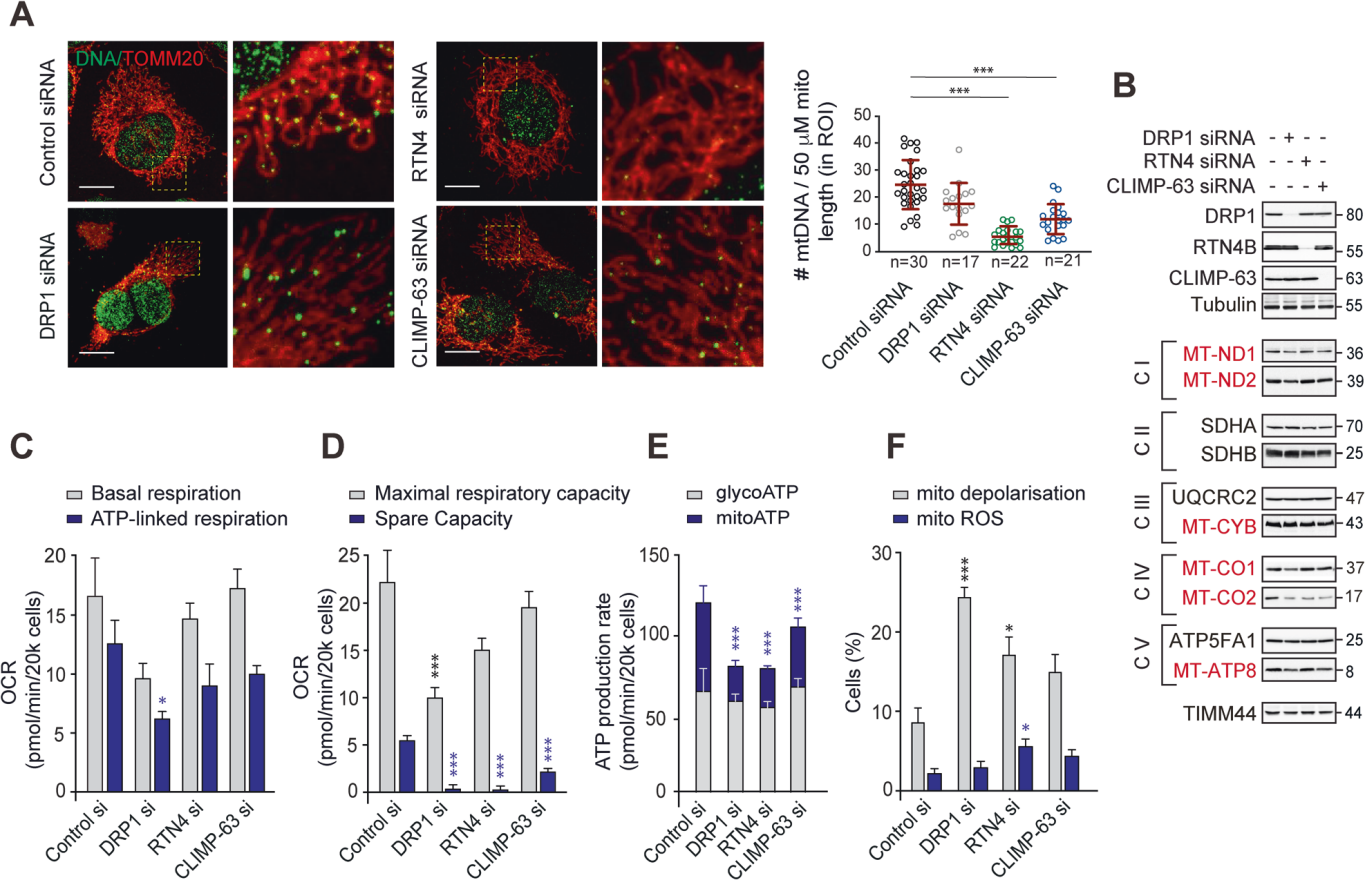
Loss of DRP1 or ER shaping proteins, RTN4 or CLIMP-63, decreases mtDNA foci, mtDNA-encoded proteins and alters different facets of mitochondrial respiration. **A** H1299 cells, transfected with control, DRP1, RTN4 or CLIMP-63 siRNA for 72 h, were fixed and immunostained against DNA (green) and TOMM20 (red). Areas demarcated by the dashed yellow boxes are magnified in the right panel of each condition. Scale bars are 10 μm. The number of mtDNA foci and mitochondrial length per ROI were calculated using ImageJ and plotted in the graph on the right. Data in are presented as mean ± SD; each dot represents one ROI within one cell, the numbers (*n*) of which (taken across three independent experiments) are indicated below the *x*-axis. ***p < 0.001 (Kruskal-Wallis test, with Dunn’s multiple comparisons test). **B** Whole cell lysates of H1299, transfected as in **A**, were immunoblotted against the indicated antibodies. mtDNA-encoded proteins are highlighted in red. **C–E** Seahorse XF-96 analysis of mitochondrial respiration in H1299 cells treated with the indicated siRNAs for 72 h prior to analysis. Histograms display quantitation of (**C**) basal *versus* ATP-linked respiration, (**D)** maximal *versus* spare respiratory capacity, and (**E**) the proportion of ATP generated from mitochondrial oxidative phosphorylation (mitoATP) *versus* that produced by glycolysis (glycoATP), both calculated using the Real-Time ATP Rate Assay. **F** Histograms display the extent of mitochondrial depolarisation and reactive oxygen species (ROS) generation in H1299 cells, transfected as in **A**. Data in **C–E** are presented as mean ± SEM, from at least 5 independent experiments. *p ⩽ 0.05, **p ⩽ 0.005, ***p ⩽ 0.001 (all ordinary one-way ANOVAs with Dunnett’s multiple comparisons tests: **C**, basal F(350) = 18.27, *p* < 0.0001; ATP-linked F(350) = 18.10, *p* < 0.0001; D, maximal F(340) = 23.19, *p* < 0.0001; spare F(337) = 15.39, *p* < 0.0001; E, mitoATP F(316) = 35.54, *p* < 0.0001; glycoATP F(316) = 2.565, *p* = 0.0910; F, depolarisation F(38) = 11.65, *p* = 0.0027; mito ROS F(316) = 3.54, *p* = 0.0388).

Since neither RTN4 nor CLIMP-63 appeared to extensively colocalise with mitochondrial DRP1 (Supplementary Fig. 8) or facilitate its recruitment (Fig. 4), we speculated that RTN4 and CLIMP-63 regulated mitochondrial hyperfusion *via* independent mechanisms, potentially involving mtDNA-containing nucleoids, a subset of which have been claimed to also mark mitochondrial constriction sites [8]. To label mtDNA, we immunostained HeLa cells using an anti-DNA antibody and counterstained mitochondrial membranes with TOMM20 (Fig. 5A). A modest decrease in the number of mtDNA-positive punctae was observed in the DRP1 downregulated cells; this reduction was much more pronounced in cells lacking RTN4 or CLIMP-63 (Fig. 5A). Although a reduction in mtDNA could imply enhanced turnover, downregulation of DRP1 also resulted in reduced expression levels of a few mtDNA-encoded proteins, all of which play integral roles in the ETC and oxidative phosphorylation (Fig. 5B and Supplementary Fig. 10). Therefore, we examined whether key parameters of mitochondrial respiration were impacted in these conditions. The basal oxygen consumption rate (OCR), ATP (synthase)-linked respiration and maximal respiratory capacity were compromised in cells lacking DRP1, but exhibited little/no change in the RTN4 and CLIMP-63 siRNA-transfected cells (Fig. 5C, D). This suggested that DRP1 may be more important than the ER shaping proteins for efficient oxidative phosphorylation to meet the ATP requirements of the cells. In contrast, the spare (reserve) capacity was largely diminished following depletion of DRP1 or the ER shaping proteins (Fig. 5D), indicating that these proteins are critical for cells to meet a sudden increase in energy demand, upon exposure to stress conditions. These effects correlated with a marked reduction in the mitochondrial ATP production with little/no impact on glycolytic ATP synthesis (Fig. 5E), confirming that DRP1, RTN4 and CLIMP-63 exclusively targeted mitochondria to regulate oxidative phosphorylation. Furthermore, in accordance with the functions of ETC complexes in the regulation of mitochondrial membrane potential [21, 22], depletion of DRP1, RTN4 or CLIMP63 enhanced mitochondrial depolarisation, with minor effects on the mitochondrial accumulation of reactive oxygen species (ROS) (Fig. 5F).

### RTN4 and CLIMP-63 differentially regulate BAK activation and BAX translocation upstream of MOMP to regulate apoptosis

**Fig. 6:**
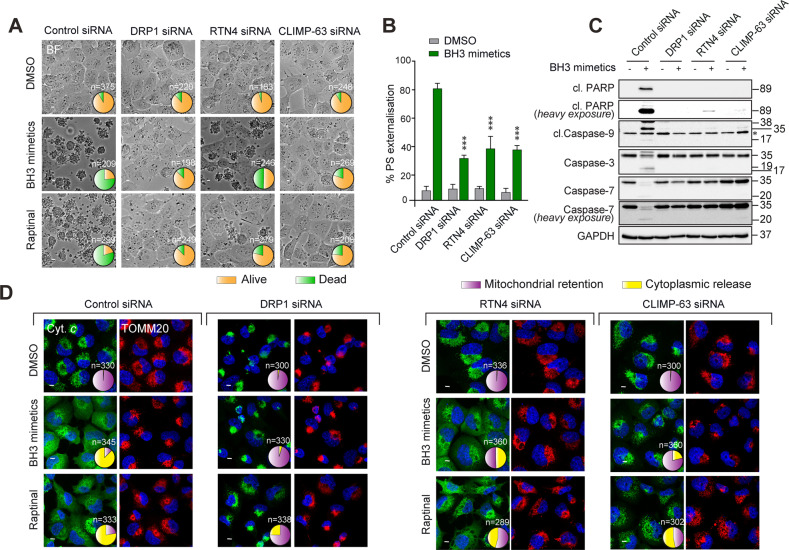
ER shaping proteins differentially regulate MOMP and apoptosis in a stimulus-specific manner. **A** H1299 cells, transfected with control, DRP1, RTN4 or CLIMP-63 siRNA for 72 h, were exposed to a combination of BH3 mimetics, A-1331852 (0.1 μM) and A-1210477 (10 μM), or Raptinal (10 μM) for 4 h. Brightfield images show live *versus* dead cells, which were counted in the specified numbers of cells (*n*) across 3 independent experiments and are presented as pie charts here, and as bar graphs in Supplementary Fig. 13. **B** Cells from (**A)** were stained with Annexin-V and PI and the extent of phosphatidylserine (PS) externalisation measured *via* flow cytometry. Data shown are mean ± SEM, from at least 3 independent experiments. ***p ⩽ 0.001 (ordinary one-way ANOVAs with Dunnett’s multiple comparisons tests; F(318) = 19.84, *p* < 0.0001). **C** Cells from (**A)** were lysed and immunoblotted for the indicated antibodies. The abbreviation cl denotes ‘cleaved’ and * in the cleaved caspase 9 blot denotes a non-specific band. **D** H1299 cells transfected with the indicated siRNAs for 72 h, were exposed to Z-VAD.fmk (30 μM) for 0.5 h, followed by a combination of BH3 mimetics, A-1331852 (0.1 μM) and A-1210477 (10 μM), or Raptinal (10 μM) for 4 h. Cells were then fixed and immunostained against cytochrome *c* (green) and TOMM20 (red). The extent of cytochrome *c* release from the mitochondria was manually counted in the indicated numbers of cells (*n*) across 3 independent experiments and the results are displayed as pie charts here, and as bar graphs in Supplementary Fig. 13. Scale bars are 10 μm.

Although silencing the expression of DRP1 perturbed mitochondrial structure, function and membrane potential, several reports have documented the anti-apoptotic role of DRP1 ablation against several apoptotic stimuli [11, 12, 23, 24]. Consistent with these reports, downregulation of DRP1 resulted in maximal protection against apoptosis induced by two distinct apoptotic stimuli: BH3 mimetics and Raptinal [25] (Fig. 6A and Supplementary Figs. 11 and 13). These anti-apoptotic effects were also observed in cells lacking CLIMP-63, and to a lesser, yet significant, extent in the RTN4-downregulated cells (Fig. 6A and Supplementary Figs. 11 and 13). These changes were accompanied by significant reductions not only in the extent of phosphatidylserine (PS) externalisation (Fig. 6B), but also caspase activation and activity (marked by the enhanced processing of the canonical caspase substrate, PARP, as well as caspases -9, -3 and -7; Fig. 6C). Moreover, downregulation of RTN4, CLIMP-63 or DRP1 also significantly reduced MOMP (characterised by the release of mitochondrial cytochrome *c*), albeit to varying extents, in cells exposed to BH3 mimetics or Raptinal (Fig. 6D and Supplementary Fig. 13), suggesting that the ER shaping proteins and DRP1 all exert their apoptotic roles upstream of MOMP.

**Fig. 7:**
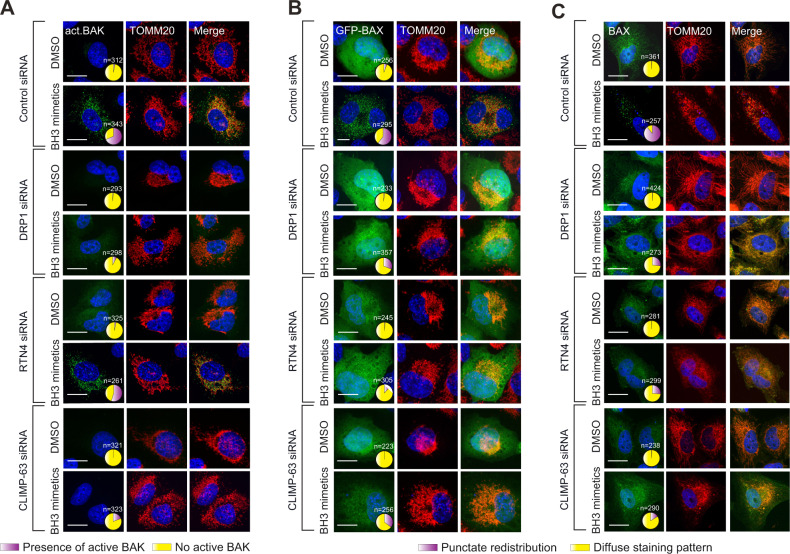
DRP1 and CLIMP-63 mediate BAK activation, and along with RTN4, regulate BAX translocation. **A** H1299 cells were transfected with control, DRP1, RTN4 or CLIMP-63 siRNA for 72 h, exposed to Z-VAD.fmk (30 μM) for 0.5 h, followed by a combination of BH3 mimetics, A-1331852 (0.1 μM) and A-1210477 (10 μM), for 4 h. Cells were then fixed and immunostained against active-BAK (green) and TOMM20 (red) and the appearance of active BAK on the mitochondrial membrane (punctate staining pattern) quantified in the indicated numbers of cells (*n*) across 3 independent experiments; the results are displayed as pie charts here, and as bar graphs in Supplementary Fig. 13. **B** H1299 cells, overexpressing GFP-BAX, transfected and treated as in (**A)**, were fixed and immunostained against TOMM20 (red) and the extent of BAX (green) redistribution to the mitochondrial membrane (punctate staining pattern) was quantified and plotted as above. **C** HeLa cells, transfected and treated as in (**A**), were fixed and immunostained against BAX (green) and TOMM20 (red) and the extent of BAX redistribution (green, punctate) quantified and plotted as above. Scale bars are 10 μm.

MOMP results from the formation of BAX/BAK oligomeric pores on mitochondrial membranes, which occurs as a consequence of BAX/BAK activation and mitochondrial translocation of BAX [14, 15]. Using an antibody that recognises the active conformation of BAK, we identified that BH3 mimetic-mediated activation of BAK was significantly diminished following the silencing of DRP1 and CLIMP-63 but not RTN4 (Fig. 7A and Supplementary Figs. 12A and 13). However, cells lacking RTN4 still exhibited significant protection against BH3 mimetic-mediated apoptosis (Fig. 6A and B), suggesting that RTN4 may act downstream of BAK activation to regulate MOMP. Therefore, we monitored the extent of mitochondrial translocation of BAX in cells lacking DRP1, RTN4 and CLIMP-63. Exposure of both H1299 (expressing Tet-inducible BAX; as these cells have no detectable endogenous BAX) and HeLa cells to BH3 mimetics resulted in a punctate redistribution of BAX, indicative of its recruitment to mitochondrial membranes (Fig. 7B and C and Supplementary Fig. 13). This redistribution was greatly reduced not only in cells lacking DRP1 or CLIMP-63, but also - and to the greatest extent - in RTN4-depleted cells (Fig. 7B, C and Supplementary Fig. 13), suggesting that DRP1 and CLIMP-63, but not RTN4, regulate BH3 mimetic-mediated BAK activation, whereas all these proteins are required for the mitochondrial translocation of BAX. In agreement, BH3 mimetic-mediated redistribution of BAX occurred not only onto mitochondrial membranes, but also to RTN4- or CLIMP-63-positive ER membranes (Supplementary Fig. 12B).

### DRP1, RTN4 and CLIMP-63 may function at distinct stages to perturb mitochondrial integrity and apoptosis

**Fig. 8:**
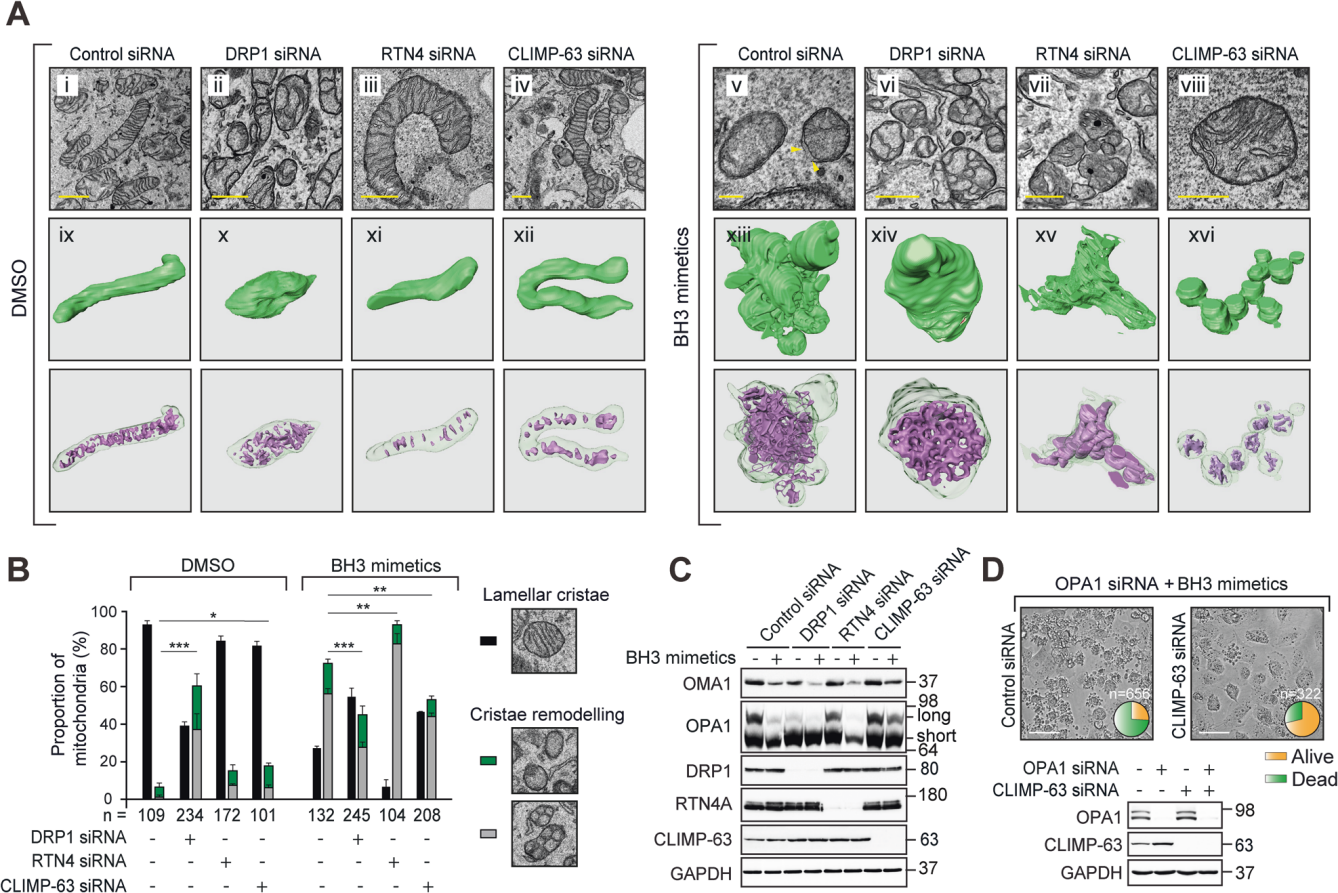
ER shaping proteins differentially regulate OPA1 proteolysis and mitochondrial cristae remodelling. **A** TEM images in H1299 cells transfected with the indicated siRNA and treated with a combination of BH3 mimetics, A-1331852 (0.1 μM) and A-1210477 (10 μM), and the corresponding renderings of isolated mitochondria (green) and cristae (purple). Scale bars are 500 nm. **B** The extent of cristae remodelling from **A** was quantified in the indicated numbers of mitochondria (*n*) across 3 independent experiments. The histograms are colour-coded to depict the extent of mitochondria possessing lamellar cristae (black), balloon-like cristae (grey) or no cristae (green), and data are presented as mean ± SEM. *p ⩽ 0.05, **p ⩽ 0.005, ***p ⩽ 0.001 (ordinary one-way ANOVAs with Dunnett’s multiple comparisons tests, for remodelled cristae only; DMSO F(38) = 118.0, *p* < 0.0001; BH3 F(38) = 49.0, *p* < 0.0001). **C** Whole cell lysates of H1299 cells, transfected with the indicated siRNA for 72 h, and exposed to Z-VAD.fmk (30 μM) for 0.5 h, followed by a combination of BH3 mimetics, A-1331852 (0.1 μM) and A-1210477 (10 μM) for 4 h, were immunoblotted against the indicated antibodies. **D** H1299 cells, transfected with siRNAs against OPA1 and/or CLIMP-63 for 72 h, were exposed to a combination of BH3 mimetics, A-1331852 (0.1 μM) and A-1210477 (10 μM) for 4 h. Brightfield images show live *versus* dead cells, which were counted in the specified numbers of cells (*n*) across 3 independent experiments and are presented as pie charts here, and as bar graphs in Supplementary Fig. 13. Western blots reveal knockdown efficiencies of the siRNAs used.

In order to further delineate the functions of DRP1, RTN4 and CLIMP-63 in the events leading up to apoptosis, we performed TEM analysis and 3View rendering of the different siRNA-transfected cells in the presence and absence of BH3 mimetics. Exposure to BH3 mimetics resulted in swollen mitochondria, that accompanied a loss of well-defined, lamellar mitochondrial cristae, as well as clear breaks in the mitochondrial outer membrane (denoted by the yellow arrow heads in Fig. 8A–v), possibly mediated by BAX/BAK pores. This was also evident when the individual mitochondria (green) and cristae (purple) were rendered from 3View microscopy, in which mitochondria appeared swollen with numerous bud-like protrusions and severely disrupted cristae (Fig. 8A–ix versus xiii). In contrast, cells deficient in DRP1, RTN4 or CLMIP-63, following exposure to BH3 mimetics, were largely intact, despite exhibiting extensive cristae remodelling, in the case of DRP1 and RTN4 downregulation (Fig. 8A–vi–viii). It must be noted that cristae remodelling was evident even in the absence of BH3 mimetics in cells transfected with DRP1 siRNA (Fig. 8A–ii and B). The hyperfused mitochondria in cells lacking RTN4 or CLIMP-63 (Fig. 8A–iii, A–iv and B) underwent cristae remodelling upon exposure to BH3 mimetics; extensively in the RTN4-, but less so in the CLIMP-63-downregulated cells (Fig. 8A–vii, A–viii and B). These findings suggested that mitochondrial cristae remodelling could be regulated primarily by CLIMP-63, with little/no influence from either RTN4 or DRP1.

Mitochondrial cristae remodelling accompanies the activation of OMA1 protease and the consequent proteolysis of OPA1 [13, 26], which in turn results in the redistribution of cytochrome *c* from the cristae to the mitochondrial intermembrane space. This can then be acted upon by DRP1-mediated mitochondrial constriction and BAX/BAK pore formation to facilitate MOMP [13, 27]. Firstly, BH3 mimetic-mediated activation of OMA1 (characterised by its degradation due to autocatalytic proteolysis) was more evident in cells lacking DRP1 and RTN4 than CLIMP-63 (Fig. 8C). This complemented BH3 mimetic-mediated proteolysis of the high molecular weight, long forms of OPA1 in cells lacking DRP1 and RTN4, but not CLIMP-63 (Fig. 8C), confirming the requirement of CLIMP-63 for OMA1 activation and OPA1 proteolysis (mitochondrial cristae remodelling) to regulate MOMP. However, the primary role of CLIMP-63 in apoptosis could not be attributed to its role in cristae remodelling, as simultaneous downregulation of OPA1 (to mimic cristae remodelling) and CLIMP-63 efficiently antagonised BH3 mimetic-mediated apoptosis (Fig. 8D), suggesting roles of CLIMP-63 downstream and/or independent of mitochondrial cristae remodelling, presumably at the level of BAK activation (Fig. 7), during apoptosis.

In summary, we have identified novel roles for two key ER shaping proteins, RTN4 and CLIMP-63, in the regulation of not only mitochondrial structure, but also critical associated processes, including respiration and apoptosis. Such roles appear to be independent of their opposing functions in maintaining ER ultrastructure, and are also distinct from that of DRP1, which is widely thought to be a master regulator of mitochondrial dynamics.

## Discussion

Although the requirement of DRP1 in the regulation of mitochondrial membrane dynamics is well established, several other proteins commonly found in the plasma membrane (Dynamin 2) [23], late endosomes/lysosomes (Rab7, VPS35) [28, 29], peroxisomes (Pex3, Pex5) [30], cytoskeleton (Septin 2, Spire1C) [31, 32], ER (INF2) [33] and the golgi complex (ARF1) [34, 35] have been recently shown to regulate mitochondrial membrane dynamics. It is also understood that ER membranes wrap around and mark mitochondrial constriction sites to which DRP1 is recruited at the onset of fission [5]. Since ER architecture comprises of structurally distinct tubules and sheets [6, 7], we postulated that a shift in dynamics from ER sheets to tubules could alter the extent of ER wrapping around the mitochondria, which in turn could impact mitochondrial fission. In this study, we report for the first time that proteins RTN4 and CLIMP-63, despite differentially regulating ER ultrastructure, demonstrated strikingly similar effects in the regulation of mitochondrial morphology (Fig. 2 and Supplementary Fig. 4), thereby suggesting that these ER shaping proteins could influence mitochondrial fission-fusion dynamics, independent of their purported functions in the maintenance of ER architecture. It is possible that the reduction in ERMCS following downregulation of DRP1, RTN4 and CLIMP-63 could contribute to mitochondrial hyperfusion (Fig. 1 and Supplementary Fig. 4).

Depletion of RTN4 and CLIMP-63 resulted in a hyperfused mitochondrial network, even in cells that are primed to undergo mitochondrial fragmentation (Fig. 3 and Supplementary Fig. 7). Furthermore, our data demonstrated the requirement of these ER shaping proteins in DRP1-mediated mitochondrial fragmentation, downstream/independent of DRP1 membrane recruitment, as downregulation of RTN4 and CLIMP-63 did not impact the translocation of DRP1 (Fig. 4). It is of course possible that RTN4 and CLIMP-63 could colocalise with DRP-1 to facilitate mitochondrial fragmentation by regulating other aspects of DRP1 activity, such as SUMOylation [36]. Nonetheless, the mitochondrial effects associated with RTN4 and CLIMP-63 depletion appear to be stimulus-specific, as our data implicate disparate roles for these proteins in OPA1 siRNA-induced mitochondrial fragmentation (Fig. 3). This could be attributed to CLIMP-63 but not RTN4 exerting its function upstream of OPA1 proteolysis (Fig. 8). Furthermore, the ability of these proteins to antagonise mitochondrial fragmentation as well as several hallmarks of apoptosis (such as BAX translocation, BAK activation, MOMP and caspase activation) may not be coupled, as mitochondrial fragmentation is not a prerequisite for MOMP and apoptosis [37], and can occur even in the absence of BAX and BAK [38].

Several studies point to a bidirectional functional link between mitochondrial dynamics and the ETC [39–42], in which inhibitors of the ETC, including Antimycin A, Rotenone and CCCP enhanced DRP1-mediated mitochondrial fission [11, 43], whereas loss of OPA1 dampened ETC and ATP production [44, 45]. Defects in mitochondrial respiration could also be attributed to the inability of cells to maintain optimal ERMCS (Figs. 1, 5 and Supplementary Fig. 4), as defects in ERMCS could impede calcium transfer between the ER and mitochondria, decrease the activity of matrix dehydrogenases, whilst inhibiting ATP synthase, thereby affecting mitochondrial respiration and ATP production [46, 47]. Furthermore, a decrease in the length and number of ERMCS in cells lacking RTN4 and CLIMP-63 correlated with a striking reduction in mtDNA-containing nucleoids, which negatively impacted the translation of mtDNA-encoded proteins, especially MT-CO2, and respiratory capacity (Fig. 5). It is indeed conceivable that reduced mitochondrial calcium uptake (as a result of reduced ERMCS or increased mitochondrial depolarisation) is partly or wholly responsible for the reduction in mitochondrial respiration. However, this hypothesis does not take into account the reduction of mtDNA foci or electron transport chain proteins following RTN4 or CLIMP63 knockdown.

In this study, we have shown that downregulation of DRP1 and the ER shaping proteins reduced not only the extent of mitochondrial fission-fusion dynamics, but also mitochondrial respiration and membrane potential (Figs. 1, 2 and 5), all of which could potentially be detrimental to the cells. However, the ability of DRP1 to influence apoptosis has been countered by its roles in invasion, metastasis and proliferation of cancer cells [48]. Similarly, DRP1 and ER shaping proteins appeared to play a more fundamental role in the induction of BH3 mimetic- and Raptinal-mediated MOMP and apoptosis (Fig. 6 and Supplementary Fig. 11). While BH3 mimetic-mediated mitochondrial translocation of BAX required DRP1, RTN4 and CLIMP-63, activation of the other pro-apoptotic effector protein, BAK occurred in a DRP1- or CLIMP-63-dependent, but RTN4-independent manner (Fig. 7 and Supplementary Fig. 12). This is in agreement with our results demonstrating the inability of RTN4 siRNA to completely reverse BH3 mimetic-mediated cytochrome *c* release, PS externalisation or membrane blebbing (Fig. 6 and Supplementary Fig. 11), which could be attributed to functional redundancy of other members of the reticulon family, namely RTN1-3 [6].

This is the first study to describe the roles of ER shaping proteins in mitochondrial membrane dynamics, energetics and cell death. Further studies are required to understand the role of ER shaping proteins in cristae remodelling and possible interactions with proteins at the OMM. Based on our findings, we hypothesise an integrative model of ER-mitochondrial membrane dynamics and BH3 mimetic-mediated apoptosis, in which BH3 mimetics facilitate the recruitment of DRP1 to mitochondrial constriction sites and induce mitochondrial fission. Meanwhile, the apoptotic signals are transduced to the inner mitochondrial membrane-resident OPA1, which undergoes OMA1-mediated proteolysis to redistribute cytochrome *c* in the mitochondrial intermembrane space. Our data demonstrate that both mitochondrial cristae remodelling and BAK activation occur in a CLIMP-63-dependent manner (Figs. 7 and 8). Although RTN4 does not appear to play any significant role in mitochondrial cristae remodelling and BAK activation, it regulates the membrane recruitment of BAX, thus facilitating the induction of BH3 mimetic-mediated apoptosis. As several cancer chemotherapeutic agents are designed to target mitochondrial structure/function, further knowledge of the interplay between the ER and mitochondria dynamics in the context of apoptosis induction will lead to more effective therapies.

## Materials and methods

### Cell culture

HeLa, MCF7, COS7 and H1299 cells were purchased from ATCC (Middlesex, UK). All cell lines were authenticated by short tandem repeat (STR) profiling and tested negative for mycoplasma. HeLa and COS7 cells were cultured in DMEM, MCF7 in EMEM and H1299 in RPMI 1640 medium. Cells were maintained at 37 °C in a humidified atmosphere of 5% CO_2_. All media were purchased from ThermoFisher Scientific (Paisley, UK) and supplemented with 10% foetal calf serum (also from ThermoFisher Scientific). Stable pools of H1299 cells expressing GFP-BAX were generated by transfecting cells with GFP-BAX-pCW plasmid, followed by selection using Puromycin (2.5 μg/mL). To induce BAX expression, the relevant cells were treated with doxycycline (1.0 μg/mL) for 48 h.

### Reagents

A-1331852, A-1210477, Z-VAD.fmk, Thapsigargin, GSK2606414, CCCP, FCCP, Oligomycin, Rotenone and Antimycin A were obtained from Selleckchem (Houston, TX, USA). Raptinal was from BioVision (Cambridge Biosciences, Cambridge, UK). Sodium Iodate and Bismaleimidohexane (BMH) were from ThermoFisher Scientific. Antibodies against Cytochrome *c* (#556432), EEA1 (#610457), Calnexin (#610524), OPA1 (#612607) and DRP1 (#611113) from BD Biosciences (San Jose, CA, USA), HSP70 (#2799), TOMM20 (#186735), RTN4 (A + B; #47085), BiP (#21685), Tubulin (#6046), PEX-14 (#183885), SDHB (#14714), UQCRC2 (#14745), MT-CO1 (#14705), MT-CO2 (#110258), ATP5FA1 (#117991), FACL4 (#137525) and Smac (#111893) from Abcam (Cambridge, UK), CLIMP-63 (#ALX-804-604) from Enzo Lifesciences (Exeter, UK), KTN-1 (#HPA003178), STX17 (#HPA001204) and TIMM44 (#HPA043052) from Sigma-Aldrich (St. Louis, MO, USA), RTN4A (#13401), Cytochrome *c* (#4272), PERK (#3192), phospho-eIF2α (#3597), MFN1 (#13196), MFN2 (#9482), MFF (#84580), DRP1^S637^ (#4867), DRP1^S616^ (#3455), SDHA (#11998), PARP (#9532), ERp57 (#2881), OMA1 (#95473), cleaved PARP (#5625), cleaved Caspase-9 (#9505), Caspase-3 (#9662) and Caspase-7 (#9494) from Cell Signaling Technology (Danvers, MA, USA), BAK (Ab-1; AM-03), DNA (#CBL186) and γH2AX (#05-636) from Merck Millipore (Dorset, UK), TRAPα (#PA5-52811) from ThermoFisher Scientific, MiD49 (#16413-1AP) and MiD51 (#20164-1-AP) from ProteinTech (Manchester, UK), MT-ND1 (#593022), MT-ND2 (#214278), MT-CYB (#335184) and MT-ATP8 (#540487) from Biorbyt (Cambridge, UK) and RTN4 (#271878), CHOP (#56107), Sig1R (#137075), Actin (#1616R) and GAPDH (#25778) from SantaCruz Biotechnology (Santa Cruz, CA, USA) were used. All other reagents, unless specified, were from Sigma.

### Plasmids

GFP-MFF was a gift from Gia Voeltz (Addgene plasmid #49153; http://n2t.net/addgene:49153; RRID: Addgene_49153) [5]. GFP-BAX and GFP-FIS1 were gifts from Dr Edward Bampton (MRC-Toxicology Unit, Leicester, UK). To clone GFP-BAX in an inducible vector, GFP-BAX was PCR-amplifed using 5′-GCTGCTAGCATGGTGAGCAAGGGCGAGGAG-3′ and 5′-GGGGCCGGCCTCAGCCCATCTTCTTCCAGATG-3′ and cloned into pCW-CAS9 plasmid (a gift from Eric Lander and David Sabatini; Addgene plasmid #50661; http://n2t.net/addgene:50661; RRID: Addgene_50661) [49], following the removal of CAS9 using restriction enzymes, NheI and FseI. mCherry-CLIMP-63, a gift from Gia Voeltz (Addgene plasmid #136293; http://n2t.net/addgene:136293; RRID: Addgene_136293) [50] was cut with NheI and EcoRI to subclone mCherry-CLIMP-63 into pLJM1 (a gift from Joshua Mendell; Addgene plasmid #91980; http://n2t.net/addgene:91980; RRID: Addgene_91980) [51]. To clone mCherry-RTN4B, pmCherry-C1 plasmid from Clontech (San Jose, CA, USA) was cut with NheI and XhoI to obtain mCherry. cDNA isolated from HeLa cells was used to amplify RTN4B using 5′-CTCCTCGAGGGATGGAAGACCTGGACCAGTCT-3′ and 5′-GAAGAATTCTCATTCAGCTTTGCGCTTCAATC-3′. Thus obtained mCherry and RTN4B were ligated with pLJM1, previously cut with NheI and EcoRI, as mentioned above. All plasmids were sequence-verified by Sanger sequencing at Source Bioscience (Nottingham, UK).

### Transfections

For RNA interference, cells were transfected with 10 nM of siRNAs against DRP1 #1 (SI04274235), DRP1 #2 (s19561), RTN4 #1 (s32767), RTN4 #2 (s32768), CLIMP-63 #1 (s225461), CLIMP-63 #2 (SI00347242), MFF (SI04374174) and OPA1 (SI03019429) purchased from either ThermoFisher Scientific or Qiagen Ltd. (Manchester, UK), using Interferin (Polyplus Transfection Inc, NY), according to the manufacturer’s protocol and processed 72 h after transfection. Both RTN4 siRNAs used in this study targeted all three isoforms (A, B and C) of RTN4, since different cell lines express either RTN4A or RTN4B, as evident from the Western blots (in Fig. 2C and Supplementary Fig. 4C). For overexpression, cells were transfected with the relevant plasmids using Lipofectamine 2000 (ThermoFisher Scientific), according to the manufacturer’s protocol and processed 24–72 h after transfection.

### Bright field imaging

Bright field images were acquired using EVOS FLOID imaging station (ThermoFisher Scientific).

### Immunofluorescence and confocal microscopy

For immunofluorescent staining, cells grown on coverslips were fixed with either 4% (w/v) paraformaldehyde and permeabilised with 0.5% (v/v) Triton X-100 in PBS, or ice-cold methanol for 5 min, followed by incubations with primary antibodies, the appropriate fluorophore-conjugated secondary antibodies, mounted on glass slides and imaged using a 3i Marianas spinning disk confocal microscope, fitted with a Plan-Apochromat 63x/1.4NA Oil Objective, M27 and a Hamamatsu ORCA-Flash4.0 v2 sCMOS Camera (all from Intelligent Imaging Innovations, GmbH, Germany). Acquired images were processed using ImageJ software (NIH, Bethesda, MD). For image quantitations, investigators were blinded as to the pre-treatment of the samples.

### Superresolution imaging

Superresolution imaging was performed on a Zeiss Elyra 7 platform with Lattice SIM (Carl Zeiss AG, Germany). Fluorophores were exited using 405, 488, 561 and 642 nm laser lines and samples were imaged using a 63×/1.4NA Oil objective. For 3D imaging, the grating pattern was used generating 13 phase images as basis for the superresolution image reconstruction. In all cases, laser power and camera exposure times were adjusted appropriately to ensure high signal to noise ratio, and visible modulation of the sample with the emission light pattern. For z-sampling, the Nyquist criterion was applied for the channel with the smallest λexc. All images were acquired with a PCO.Edge 4.2 scientific complementary metal-oxide semiconductor (sCMOS) camera with exposure times ranging between 200 and 250 ms. Raw images were reconstructed using the appropriate tool of Zeiss Zen software (Black version 3.0 with Structured Illumination module) adjusting settings appropriately to avoid commonly occurring artefacts such as ringing and honeycomb background. As a rule of thumb, the best fit parameter was set with a Wiener filter for sharpness of 6.0.

### Transmission and Scanning Electron microscopy

For transmission electron microscopy, cells prepared as previously described [37] were imaged using a MegaViewIII CCD camera and AnalySIS software (EMSIS GmbH, Germany) in a FEI Tecnai G2 Spirit BioTWIN electron microscope. For Serial block face scanning electron microscopy (3View microscopy), cells were fixed in 2.5% (w/v) glutaraldehyde with 2 mM calcium chloride in 0.1 M cacodylate buffer (pH 7.4). Heavy metal staining consisted of reduced osmium (2% (w/v) OsO_4_, 1.5% (w/v) potassium ferrocyanide in ddH_2_O), 1% (w/v) thiocarbohydrazide (RT), 2% OsO_4_ (w/v in ddH_2_O), then 1% (w/v) aqueous uranyl acetate overnight at 4 °C. Cells were stained the following day with Walton’s lead aspartate (0.02 M lead nitrate, 0.03 M aspartic acid, pH 5.5) at RT. Fixation and staining steps were performed in a Pelco Biowave®Pro (Ted Pella Inc., Redding, CA) at 100 w 20 Hg, for 3 min and 1 min, respectively. Dehydration was performed in a graded series of ethanol before filtration and embedding in hard premix resin (TAAB, Reading, UK). Images were acquired using Gatan 3View serial block-face system (Gatan, Pleaseanton, CA) installed on a FEI Quanta 250 FEG scanning electron microscope (FEI Company, Hillsboro, OR). Original images with pixel sizes of 4.3–6 nm (depending on the size of the cell) were double binned in the x and y dimensions only, to reach maximum pixel sizes of 8.6–12 nm. Each z-section had a thickness of 75 nm. ERMCS were classified as contact points between ER and mitochondrial membranes separated by a distance of ≤ 25 nm, which is more stringent than the 80 nm distance that has been used previously [52, 53]. Images were processed, analysed and quantified using Amira Software 6, according to the manufacturer’s protocol (ThermoFisher Scientific).

### Bioenergetics measurements

Cells were plated in a XF96-well cell culture plates (Agilent, Santa Clara, CA, USA) at a density of 20,000 cells per well. The Mitochondrial Stress Test and Real-Time ATP Rate Assay were performed independently in a Seahorse XF Analyzer (Agilent Technologies, Santa Clara, CA), as recommended by the vendor. Briefly, H1299 cells were incubated with unbuffered Seahorse XF Base medium supplemented with 10 mM glucose, 2 mM L-glutamine, 1 mM sodium pyruvate and adjusted to pH 7.4. Cells were incubated for 1 h in a non-CO_2_ incubator prior to sequential injections of Mitochondrial Stress or ATP Test compounds. For the Mitochondrial Stress Test, XFe96 cartridge ports were loaded with oligomycin (1 µM), FCCP (2 µM) and a combination of rotenone and antimycin-A (both at 0.5 µM), all diluted in supplemented Seahorse XF Base medium. For the ATP Rate Assay, FCCP was excluded. Results obtained were normalised for total protein content, using a standard BCA assay and normalised data analysed using the Seahorse XF Mitochondrial Stress and ATP Test generator from Agilent.

### Flow cytometry

For measuring mitochondrial depolarisation and ROS accumulation, cells were trypsinised, washed with PBS, resuspended in cell culture medium along with TMRE (200 nM) and MitoSoxRed (500 nM), respectively. Following a 10 min incubation at 37 °C, samples were analysed by flow cytometry using an Attune NxT flow cytometer (ThermoFisher Scientific). For measuring the extent of phosphatidylserine externalisation, cells were trypsinised, washed with PBS, resuspended in annexin-binding buffer (10 mM HEPES, pH7.4, 5 mM KCl, 130 mM NaCl, 1 mM MgCl_2_ and 1.8 mM CaCl_2_) along with Annexin-V (made in-house; conjugated with FITC) for 8 min, followed by Propidium iodide (PI; 200 ng/mL; Sigma) for a further 2 min. Samples were analysed by flow cytometry as stated above.

### Subcellular fractionation, MAM isolation and western blotting

For fractionation of the cytoplasmic and mitochondrial fractions (Fig. 4C), cells were resuspended in 0.5 mL of the mitochondrial fractionation buffer (20 mM HEPES, pH7.4, 10 mM KCl, 2 mM MgCl_2_, 1 mM EDTA, 1 mM EGTA, 1 mM DTT and Protease inhibitor cocktail) and incubated on ice for 15 min. Cells were then lysed by passing the cell suspension through a 27 G needle, repeatedly until all cells were lysed. The lysed cells were incubated on ice for a further 20 min, followed by centrifugation at 750 x *g* for 5 min. The pellet containing the nuclear fraction was discarded, whereas the supernatant containing the cytoplasm and mitochondrial fractions was centrifuged at 10,000 x *g* for 5 min to separate the pellet (mitochondrial fraction) and supernatant (cytoplasmic fraction). The pellet was then resuspended in Tris-buffered saline (200 mM Tris and 1.5 M NaCl) containing 0.1 % SDS and sonicated to shear mitochondrial DNA. Isolation of MAM from cell lysates (Supplementary Fig. 9) was carried out as previously described [54]. Protein estimations from the different subcellular fractions were then made to proceed with SDS-PAGE electrophoresis, performed according to standard protocols. Subsequently proteins were transferred to nitrocellulose membrane and protein bands visualised with ECL reagents (GE Healthcare, Chalfont St. Giles, UK). Densitometry analyses were performed using ImageJ software. Full length Western blots are available as Supplementary Material.

### Statistical analysis

Statistical analyses were conducted using either a one-way ANOVA with Dunnett’s multiple comparisons test (Figs. 5C–F, 6B, 8B, Supplementary Figs. 10, 12A), two-way ANOVA with Bonferroni’s or Dunnett’s multiple comparisons test (Supplementary Fig. 13), multiple unpaired *t* tests with two-stage linear step-up procedure of Benjamini, Krieger, and Yekutieli (Supplementary Fig. 13), Mann-Whitney *U* test (Fig. 1D, F and Supplementary Fig. 1A), or Kruskal-Wallis test with Dunn’s multiple comparisons test (Figs. 2A, C, E, 4B, 5A, Supplementary Figs. 4B, E and 6B), to evaluate differences between different categories. Asterisks correspond to the following *p* values: *p ≤ 0.05, **p ≤ 0.005 and ***p ≤ 0.001. Sample sizes are quoted in figures/legends, and correspond to a minimum of 3 biological replicate experiments.

## Supplementary information

Reproducibility checklist

Supplementary Figure Legends

S1

S2

S3

S4

S5

S6

S7

S8

S9

S10

S11

S12

S13

Original Data File

## Data Availability

All data generated or analysed during this study are included in this published article (and its supplementary information files). Further information is available from the corresponding author on reasonable request.
