# Supplementary material for: Novel roles of RTN4 and CLIMP-63 in regulating mitochondrial structure, bioenergetics and apoptosis
Source: Cell Death Dis. 2022 May 4;13(5):436. doi: 10.1038/s41419-022-04869-8 (PMC9068774; doi:10.1038/s41419-022-04869-8)
Supplement: Supplementary file 2 — Supplementary Figure Legends [file 41419_2022_4869_MOESM2_ESM.docx]

**Supplementary Figure 1. DRP1 depletion alters mitochondrial morphology in multiple cell lines. (A)** Confocal microscopy images show mitochondrial morphology, assessed by HSP70 immunostaining (green) of HeLa, MCF7, COS7 and H1299 cells, transfected with a control or DRP1 siRNA for 72 h. Scale bars are 10 μm. Dot plots demonstrate the mitochondrial length (in μm) and number, in one region of interest (ROI; depicted by the yellow dashed boxes within the confocal images) per cell and the indicated number of cells (n) per condition; each dot represents one ROI. Data are presented as mean ± SD, ***p⩽0.001 (Mann-Whitney *U* test). **(B)** Western blots show DRP1 knockdown efficiencies in the four cell lines. DRP-1 appears as a doublet in these cell lines, suggesting potential post-translational modifications, including phosphorylation.

**Supplementary Figure 2. Serial block-face transmission electron microscopic images reveal how organelles contact each other.** The endoplasmic reticulum (red) wrapping around the chosen mitochondrion (in light blue, taken from the control siRNA-transfected H1299 cells in **Fig.** **1C**), along with the ER-mitochondria contact sites (ERMCS; red dots) juxtaposed on the mitochondrial skeleton (translucent blue) were rendered to estimate the volume (in μm^3^) of the rendered mitochondria (V_mito2_), as well as the number of ERMCS, both absolute (n_ERMCS_) and normalised to the volume of the corresponding mitochondrion (n_ERMCS_ / V_mito2_).

**Supplementary Figure 3. ER stress and the Unfolded Protein Response are not associated with mitochondrial hyperfusion following downregulation of DRP1, RTN4 or CLIMP-63. (A)** Western blots show no induction of ER stress and the UPR in HeLa cells lacking DRP1, RTN4 or CLIMP-63. Cells treated with Thapsigargin (10 μM) for 48 h was used as a positive control for UPR induction and the PERK inhibitor, GSK2606414 (1 μM) was administered for 48 h in the indicated lanes. * in the PERK blot indicates the shift in PERK due to phosphorylation, which is indicative of the UPR. **(B)** HeLa cells, transfected with a non-targeting control, DRP1, RTN4 or CLIMP63 siRNA for 72 h, and treated with either DMSO or GSK2606414 (1 μM), 24 h after transfection, were immunostained against TOMM20 (green) to visualise mitochondrial structure. Mitochondrial morphologies were qualified in the indicated numbers of cells (n) per condition, across a minimum of 3 independent experiments, and the data plotted in pie charts here, and as bar graphs in Supplementary Fig. 13. Scale bars are 10 μm.

**Supplementary Figure 4. Depletion of ER shaping proteins, RTN4 and CLIMP-63 also alters mitochondrial morphology and ERMCS.** (**A**) MCF7, COS7 and H1299 cells were transfected with a non-targeting control, DRP1, RTN4 or CLIMP-63 siRNA for 72 h, then immunostained for HSP70 (green) to visualise mitochondrial structure. Mitochondrial morphologies were qualified in the indicated numbers of cells (n) per condition, across a minimum of 3 independent experiments, and the data plotted in pie charts here, and as bar graphs in Supplementary Fig. 13. Scale bars are 10 μm. **(B)** Mitochondrial length (in μm) and number were quantified from confocal microscopy images; one region of interest (ROI) per cell, and the indicated number of cells (n) per condition. **(C)** Western blots show DRP1, RTN4 and CLIMP-63 knockdown efficiencies for the indicated siRNAs. Cell lines expressing either RTN4A or RTN4B have been probed for the relevant isoforms. **(D)** TEM images of mitochondria in H1299 cells, transfected with the indicated siRNAs for 72 h. Scale bars are 500 nm. **(E)** ERMCS were quantified in H1299 cells, transfected with the indicated siRNAs in **D**. Dot plots, from left to right, show the average number of ERMCS per mitochondrion, the absolute lengths of ERMCS (in μm) and length of ERMCS as a proportion of the mitochondrial perimeter (in %). In the first plot, each dot represents one cell; in the two right plots, each dot represents one ERMCS, and these values are presented as n in the X-axes. All numbers were generated from 10 cells per siRNA, across 3 independent experiments, and the data presented as mean ± SD, **p⩽0.005, ***p⩽0.001 (Kruskal-Wallis test, with Dunn’s multiple comparisons test).

**Supplementary Figure 5. The ability of DRP1, RTN4 and CLIMP-63 to regulate mitochondrial fusion does not accompany changes in the total levels of mitochondrial/ ER resident proteins or mitochondrial fission/fusion machinery.** Western blots of mitochondrial and ER proteins in HeLa **(A)** and COS7 **(B)** cells, following transfection with the indicated siRNAs, to examine changes in organelle mass.

**Supplementary Figure 6. DRP1 and the ER shaping proteins, RTN4 and CLIMP-63 appear to exhibit varying effects on peroxisomes.** (**A**) HeLa cells, transfected with a non-targeting control, DRP1, RTN4 or CLIMP63 siRNA for 72 h, were immunostained using antibodies against HSP70 (mitochondria; red), PEX-14 (peroxisomes; cyan) and EEA1 (early endosomes; green). Mitochondrial morphologies were qualified in the indicated numbers of cells (n) per condition, across a minimum of 3 independent experiments, and the data plotted in pie charts here, and as bar graphs in Supplementary Fig. 13. Areas outlined by the dashed yellow boxes are magnified in images 1-4: 1, EEA1; 2, HSP70; 3, PEX14; and 4, all three merged. Scale bars are 10 μm. (**B**) Peroxisomal number and peroxisomal length (in μm) were quantified in one region of interest (ROI) per cell, in the indicated numbers (n) of HeLa cells per knockdown condition. In the graphs, each dot represents one cell. (**C**) Western blots show endosome and peroxisome organelle masses for the indicated siRNAs in HeLa cells.

**Supplementary Figure 7. DRP-1 and ER shaping proteins, RTN4 and CLIMP-63, are required for MFF- and FIS1-mediated mitochondrial fragmentation.** H1299 cells, transfected with a non-targeting control, DRP1, RTN4 or CLIMP-63 siRNA for 72 h, were also transfected with either a GFP, GFP-MFF or GFP-FIS1 plasmid for the final 24 h, then immunostained using an antibody against TOMM20 (red). Mitochondrial morphologies were qualified in the indicated numbers of cells (n) per condition, across a minimum of 3 independent experiments, and the data plotted in pie charts here, and as bar graphs in Supplementary Fig. 13. Scale bars are 10 μm.

**Supplementary Figure 8. Overexpression of RTN4 or CLIMP-63 reverses the effect of the respective siRNAs against FIS1-mediated mitochondrial fragmentation.** Rescue experiments were performed, in which HeLa cells, transfected with siRNAs against RTN4 or CLIMP-63 for 72 h, were subsequently transfected with plasmids expressing GFP-FIS1, along with either (**A**) mCherry-RTN4B or (**B**) mCherry-CLIMP-63 for the final 24 h. Cells were then immunostained using an antibody against TOMM20 (cyan) and mitochondrial morphologies quantified for the indicated numbers (n) of cells, across a minimum of 3 independent experiments, and displayed as pie charts here, and as bar graphs in Supplementary Fig. 13. Scale bars are 10 μm.

**Supplementary Figure 9. RTN4 and CLIMP-63 preferentially localise to the ERMCS.** H1299 cells were lysed and fractionated to isolate fractions enriched in nucleus, mitochondria, mitochondria-associated membranes (MAM; C, crude, and P, purified), microsomes and cytosol. The fractions were then probed against antibodies for DRP1, RTN4B and CLIMP-63, along with positive controls (markers for nucleus, mitochondria, MAM, microsomes and cytosol) to demonstrate the efficiency of fractionation.

**Supplementary Figure 10. Densitometry analyses reveal a loss of mtDNA-encoded proteins following downregulation of DRP1 and the ER shaping proteins.** Bar graphs depict the densitometry analyses performed from Western blots of H1299 cells, transfected with control, DRP1, RTN4 or CLIMP-63 siRNA for 72 h and immunoblotted for the indicated proteins. Data are presented as mean ± SEM, from 3 independent experiments. **p*⩽0.05, ***p*⩽0.005 (ordinary one-way ANOVAs with Dunnett’s multiple comparisons tests: MT-ND1 F(3,8)=6.560, *p*=0.0150; MT-ND2 F(3,8)=11.26, *p*=0.0030; SDHA F(3,8)=3.566, *p*=0.0668; SDHB F(3,8)=1.473, *p*=0.2934; UQCRC2 F(3,8)=1.755, *p*=0.2332; MT-CYB F(3,8)=5.317, *p*=0.0262; MT-CO1 F(3,8)=3.094, *p*=0.0895; MT-CO2 F(3,8)=4.610, *p*=0.0373; ATP5FA1 F(3,8)=1.124, *p*=0.3953; MT-ATP8 F(3,8)=12.49, *p*=0.0022).

**Supplementary Figure 11. DRP1 and the ER shaping proteins antagonise BH3 mimetic- and Raptinal-mediated apoptosis in several cell lines.** HeLa (**A**) or MCF7 cells (**B**), transfected with control, DRP1, RTN4 or CLIMP-63 siRNA for 72 h, were exposed to a combination of BH3 mimetics, A-1331852 (0.1 μM) and A-1210477 (10 μM), or Raptinal (10 μM) for 4 h. Brightfield images show live *versus* dead cells, which were counted in the specified numbers of cells (n) across 3 independent experiments and are presented as pie charts here, and as bar graphs in Supplementary Fig. 13. Scale bars are 10 μm.

**Supplementary Figure 12. DRP1, RTN4 and CLIMP-63 co-localise with the membrane-translocated BAX during apoptosis.** (**A**) H1299 cells were transfected with control, DRP1, RTN4 or CLIMP-63 siRNA for 72 h, exposed to Z-VAD.fmk (30 μM) for 0.5 h, followed by a combination of BH3 mimetics, A-1331852 (0.1 μM) and A-1210477 (10 μM), for 4 h. Cells were then fixed and immunostained against active-BAK and the extent of BAK activation assessed by flow cytometry. Data are presented as mean ± SEM, from 4 independent experiments. **p*⩽0.05, ****p*⩽0.001 (ordinary one-way ANOVAs with Dunnett’s multiple comparisons tests: DMSO F(3,12)=3.507, *p*=0.0494; BH3 F(3,12)=49.05, *p*<0.0001). (**B**) HeLa cells were exposed to Z-VAD.fmk (30 μM) for 0.5 h, followed by a combination of BH3 mimetics, A-1331852 (0.1 μM) and A-1210477 (10 μM) for 2.5 h. Cells were then immunostained against RTN4B (R4B; yellow), BAX (magenta), CLIMP-63 (C63; blue), and TOMM20 (T20; green) and subjected to superresolution microscopy. Scale bars are 10 μm.

**Supplementary Figure 13. Bar graphs of all the pie charts shown in the indicated figures.**

Data are presented as mean ± SEM, from 3 independent experiments. **p*⩽0.05, ***p*⩽0.005, ****p*⩽0.001 (Figures 1A, S8A, S8B, two-way ANOVAs with Bonferroni’s multiple comparisons test; Figures 2B, 3A, 3B, 4A, 6A, 6D, 7A, 7B, 7C, S3A, S4A, S6A, S7, S12A, S12B, two-way ANOVAs with Dunnett’s multiple comparisons test; Figure 8D, multiple unpaired t tests with two-stage linear step-up procedure of Benjamini, Krieger, and Yekutieli).
