# Supplementary figures and images for: Novel roles of RTN4 and CLIMP-63 in regulating mitochondrial structure, bioenergetics and apoptosis
Source: Cell Death Dis. 2022 May 4;13(5):436. doi: 10.1038/s41419-022-04869-8 (PMC9068774; doi:10.1038/s41419-022-04869-8)

# Figure S1

## A

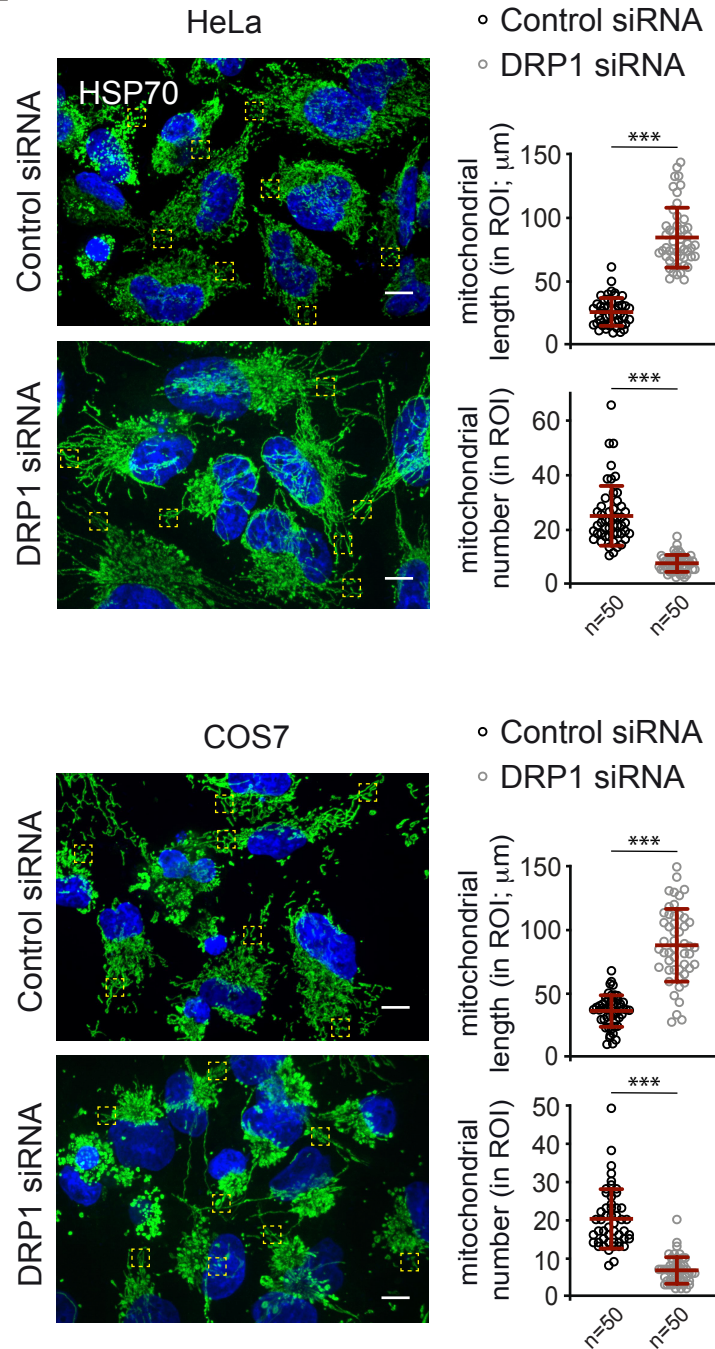

**MCF7**

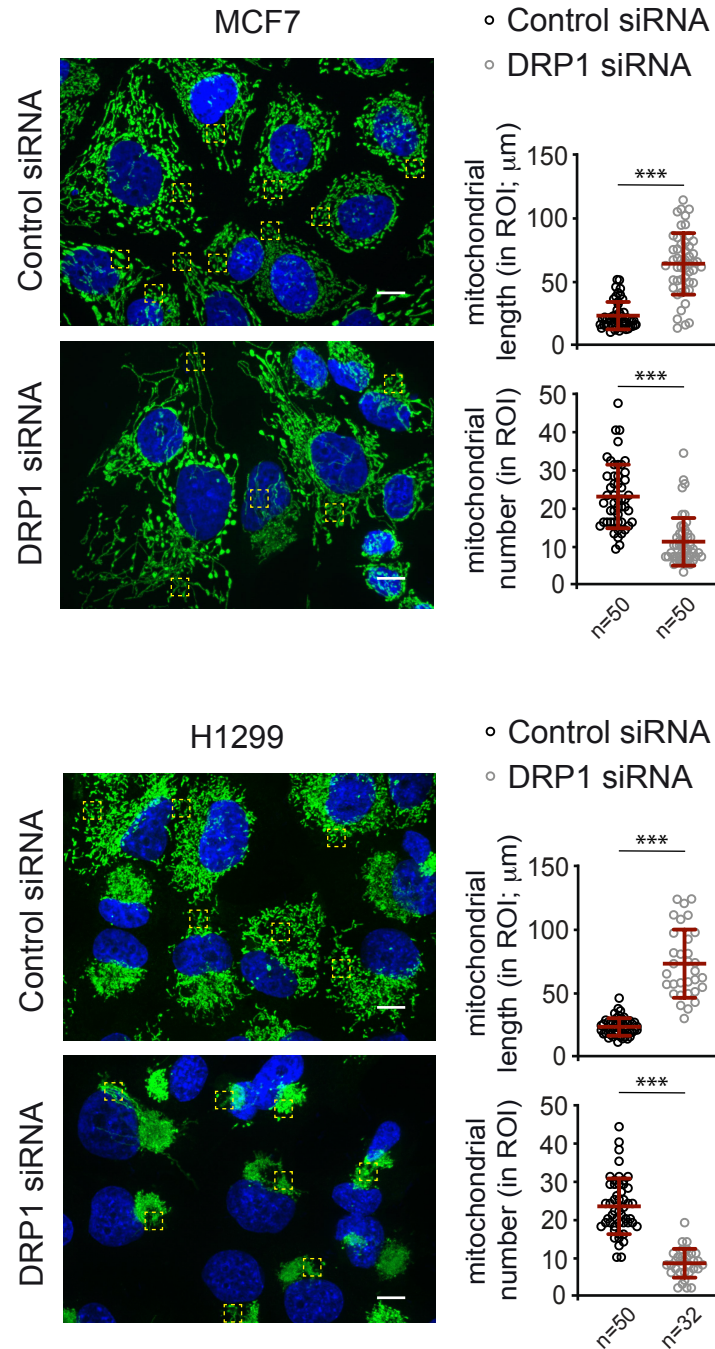

## B

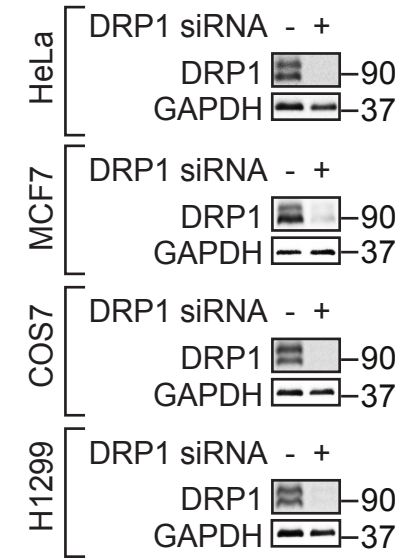

Supplement: Supplementary file 3 — S1 [file 41419_2022_4869_MOESM3_ESM.pdf]

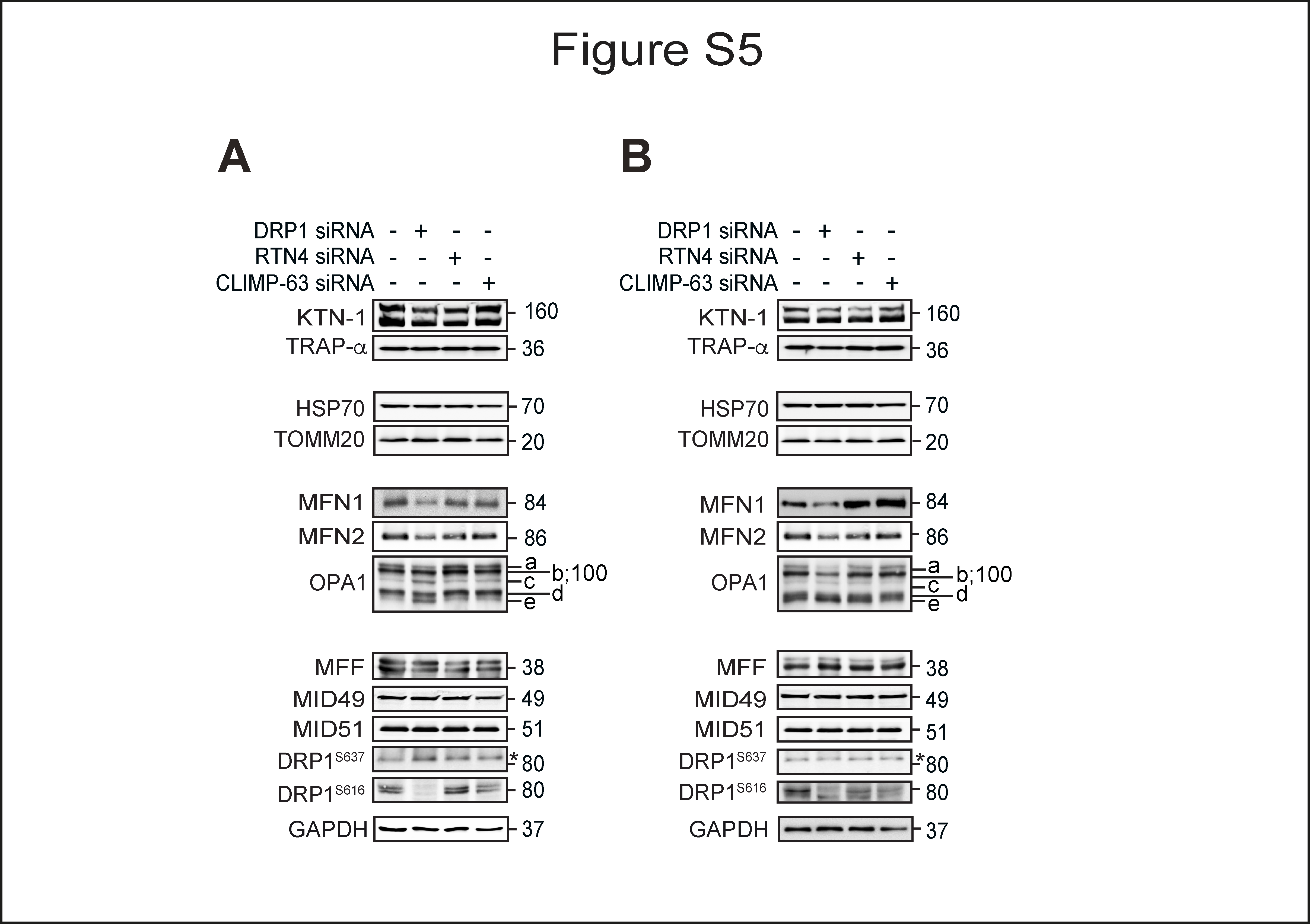

Supplement: Supplementary file 7 — S5 [file 41419_2022_4869_MOESM7_ESM.tif]

Figure S10

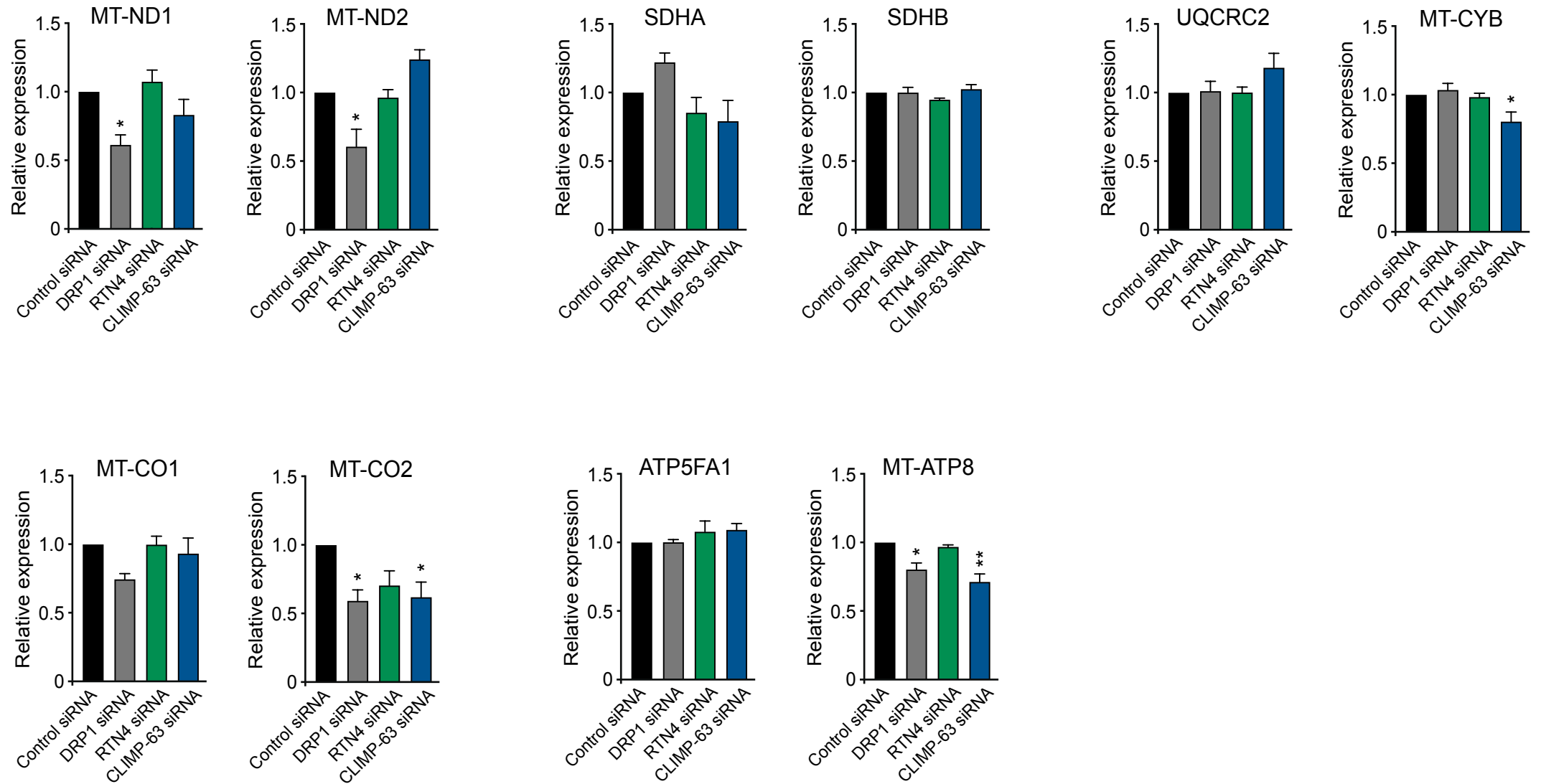

Supplement: Supplementary file 12 — S10 [file 41419_2022_4869_MOESM12_ESM.pdf]

# Figure S13

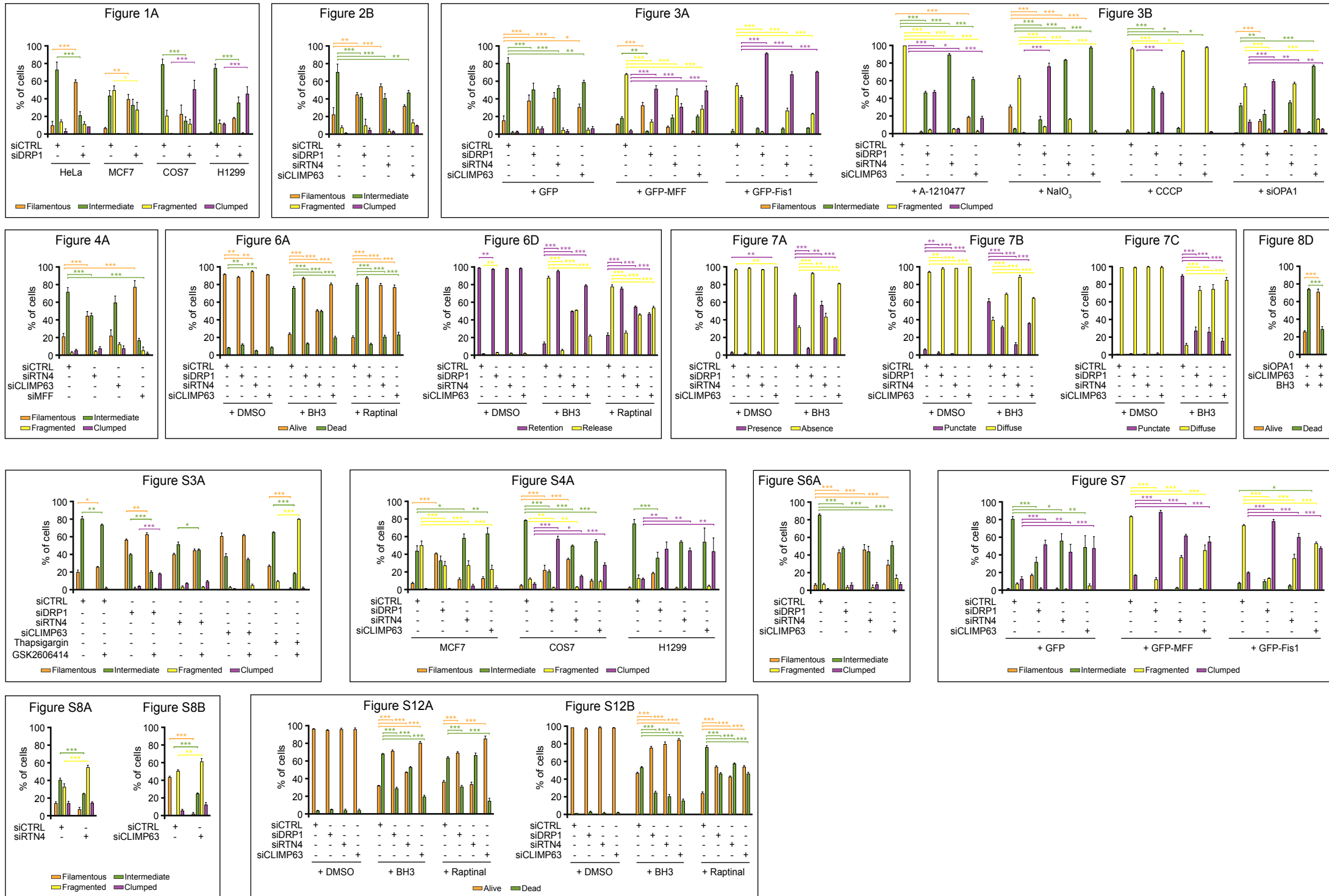

Supplement: Supplementary file 15 — S13 [file 41419_2022_4869_MOESM15_ESM.pdf]

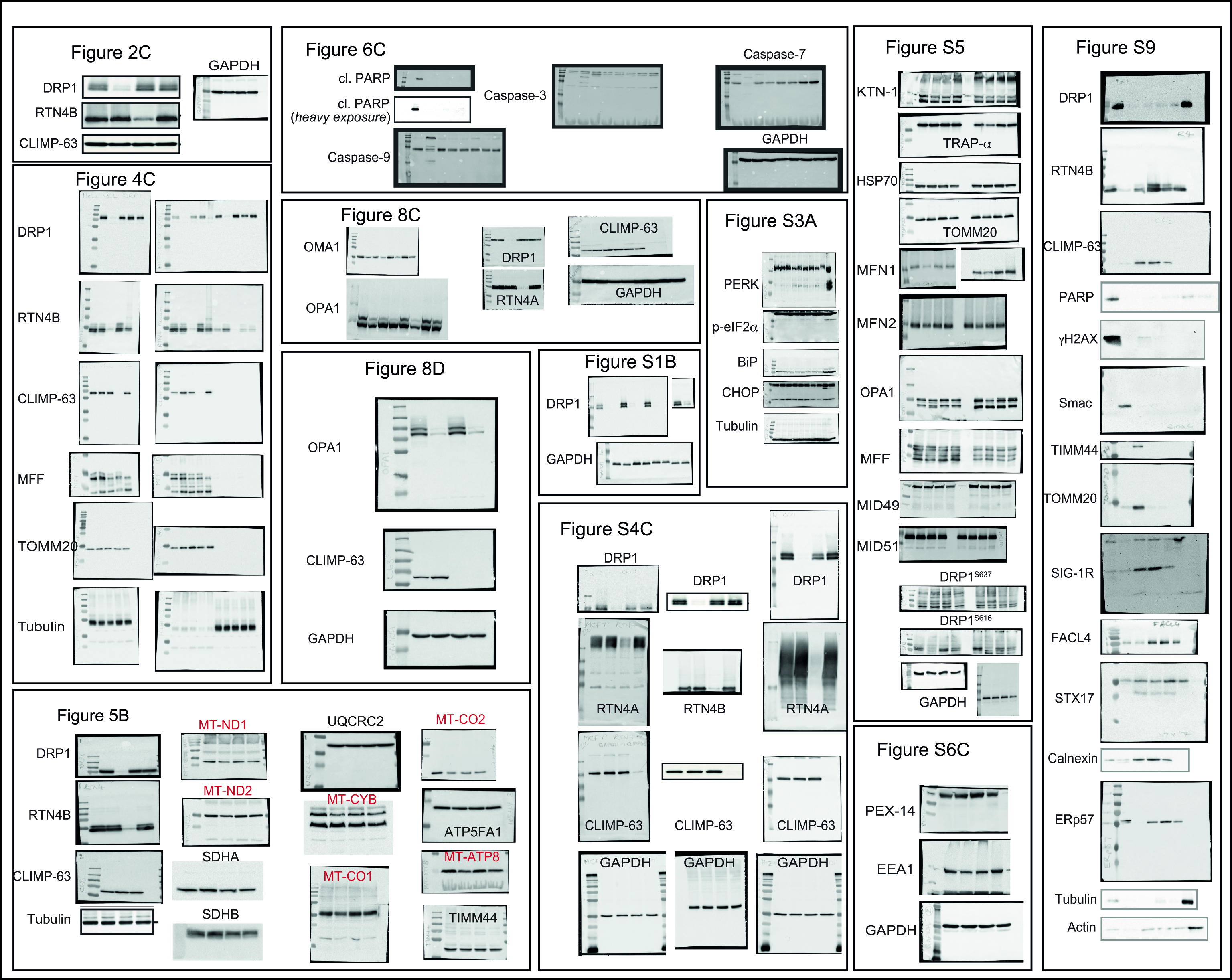

Supplement: Supplementary file 16 — Original Data File [file 41419_2022_4869_MOESM16_ESM.tif]
